# An Ultra-Wideband Frequency System for Non-Destructive Root Imaging

**DOI:** 10.3390/s18082438

**Published:** 2018-07-26

**Authors:** Thomas Truong, Anh Dinh, Khan Wahid

**Affiliations:** Department of Electrical and Computer Engineering, University of Saskatchewan, Saskatoon, SK S7N-5A9, Canada; anh.dinh@usask.ca (A.D.); khan.wahid@usask.ca (K.W.)

**Keywords:** ultra-wideband, radar imaging, image processing, delay-and-sum beamforming, non-destructive root imaging

## Abstract

Understanding the root system architecture of plants as they develop is critical for increasing crop yields through plant phenotyping, and ultra-wideband imaging systems have shown potential as a portable, low-cost solution to non-destructive imaging root system architectures. This paper presents the design, implementation, and analysis of an ultra-wideband imaging system for use in imaging potted plant root system architectures. The proposed system is separated into three main subsystems: a Data Acquisition module, a Data Processing module, and an Image Processing and Analysis module. The Data Acquisition module consists of simulated and experimental implementations of a non-contact synthetic aperture radar system to measure ultra-wideband signal reflections from concealed scattering objects in a pot containing soil. The Data Processing module is responsible for interpreting the measured ultra-wideband signals and producing an image using a delay-and-sum beamforming algorithm. The Image Processing and Analysis module is responsible for improving image quality and measuring root depth and average root diameter in an unsupervised manner. The Image Processing and Analysis module uses a modified top-hat transformation alongside quantization methods based on energy distributions in the image to isolate the surface of the imaged root. Altogether, the proposed subsystems are capable of imaging and measuring concealed taproot system architectures with controlled soil conditions; however, the performance of the system is highly dependent on knowledge of the soil conditions. Smaller roots in difficult imaging conditions require future work into understanding and compensating for unwanted noise. Ultimately, this paper sought to provide insight into improving imaging quality of ultra-wideband (UWB) imaging systems for plant root imaging for other works to be followed.

## 1. Introduction

Maximizing crop production yield is critical for meeting global crop demands and maintaining global food security [1,2,3,4,5]. Unfavorable environmental conditions are responsible for a large crop production deficit in recent years [4,6]. The method of selective breeding to create environmentally stress tolerant crops has been the most effective method for maximizing crop yields for the last half century [3]. Selective breeding methods rely on the analysis of the gene-environment interactions which are exhibited through physical characteristics in the plants [7]. The process of collecting and analyzing environmental responses and physical characteristics is called plant phenotyping. Unfortunately, effective plant phenotyping requires large quantities of accurate environmental and plant data [3]. The lack of reliable information on growing environments and individual plant measurements has bottlenecked advancements in improving crop yield [7,8]. Narrowing the issue further, the non-destructive collection of accurate data on roots, a complicated organ that is critical in the development of the plant, has proven to be a significant challenge that severely inhibits plant phenotyping research [9,10,11,12,13]. The challenge of characterizing plant roots comes with the hidden nature of the roots as they are concealed by the medium they are grown in, often needing destructive methods of measuring root development [14]. As such, a non-destructive device and imaging algorithm which measures the characteristics of plant roots would greatly benefit the field of plant phenotyping. 

Healthy roots are critical to the development and productivity of a plant on numerous levels. Roots bring in water and nutrients, store essential resources, and anchor the plant to the growing medium [15]. The term root system architecture (RSA) is used to describe the spatial distribution of the root within the growing medium. The RSA is highly dynamic and knowledge of the RSA as it develops is crucial for understanding how different root traits can benefit a plant’s development and productivity [16]. Due to the root’s role in plant development, plant scientists are interested in being able to non-destructively measure and determine crucial characteristics in the RSA in order to breed optimally productive crops which respond well to various environmental stresses. For example, characteristics such as primary root length [17] and root diameter [18] determine how much access the plant has to stored water and how well the plant can penetrate harder growing mediums. The deeper the root, the better the access and the larger the diameter, the better the penetration. Plant phenotyping allows researchers and crop field managers to understand which breeds have favorable root characteristics for the environment in which they are being grown, to maximize crop productivity [3]. Being able to non-destructively image the RSA and to measure characteristics like root depth and root diameter becomes essential for plant phenotyping.

In the past, methods for root measurements were labor-intensive and highly destructive, which often inhibited the development of the roots. A common method was a root excavation, which required physically removal of the plant from its growing medium to measure characteristics [12]. The cumbersome and destructive nature of this method made it difficult for researchers to properly record and analyze the growth of the roots. More recently, methods utilizing advanced imaging techniques such as magnetic resonance imaging (MRI) [19], X-ray computed tomography (CT) [20], positron emission tomography (PET) [21], and ultra-wideband imaging [9,22] have been in development for use in root phenotyping research. 

MRI is a common imaging technique used in the medical field to detect concealed structures in the human body with high resolution. It only follows that MRI has found use in researching plant roots. It has been used to research the effect of pot size on root structure [23] and the effect of disease on sugar beet roots [19]. Unfortunately, MRI is not feasible for many places for use high throughput root phenotyping. The associated costs and the bulkiness of the equipment restricts the ability of MRI technology to be used in plant phenotyping. Studies often required expensive rental time on third party MRI machines. Moreover, MRI has difficulties imaging roots uncontrolled soil conditions [10,13].

X-ray CT produces non-destructive 3D root images by measuring the interactions of high energy electromagnetic waves with the system to be imaged [12]. X-ray CT is capable of delivering very high quality models of the RSA for measurement and analysis [20,24,25]. Similar to MRI, X-ray CT is encumbered by needing the use of expensive and bulky scanning equipment, making it unsuitable for use in many facilities.

PET is a technique also often used in the medical field to detect concealed structures. PET images are produced by detecting positrons emitted by a radioactive isotype which is injected to the system to be scanned [11]. This results in very high quality images of roots, and can be used in conjunction with other imaging techniques to acquire complementary information [11,21]. MRI-PET imaging techniques are continuing to be developed for both plant phenotyping and medical imaging [12]. Once again, much like the other medical imaging methods, PET also requires the use of expensive equipment and interferes with the plant growth by needing to prepare the plant with radioactive isotopes.

Ultra-wideband (UWB) devices have been increasingly popular for imaging concealed structures. The compact form and low-cost of ultra-wideband equipment relative to the equipment required for MRI, X-Ray CT, and PET makes UWB an attractive option for potential use in root phenotyping purposes [26]. UWB technology uses a broad frequency spectrum to image materials [9,27]. In the time domain, this spectrum is produced by a short pulse, generally with energy concentrated in a 1 ns timeframe for GHz frequencies [26]. UWB radar involves transmitting a UWB pulse through the system under testing, and receiving and processing the reflected/transmitted response from the system under testing to detect the presence of concealed objects, similar to conventional radar. There are many design choices involved in UWB radar, such as operating frequencies, number of transmitters/receivers, and transmitter/receiver configurations. Regardless of set-up, UWB radar focuses on measuring reflectance/transmittance of UWB signals from a system under test. These measurements can then be used to generate images of concealed objects within the system under test. Like conventional radar, UWB radar needs compensation systems that deal with unwanted wave phenomena. As such, this paper presents the design, implementation, and analysis of an UWB frequency system for use in imaging concealed plant roots in potted plants. Section 2 covers the materials and methods of the UWB root imaging system, covering important design choices and implementation details. Section 3 covers the results and justification for the design decisions detailed in Section 2. Section 3 also presents the results from the experimental trials. Section 4 provides insight into the results achieved in the simulation and experimental trials of the designed system. 

## 2. Materials and Methods

**Figure 1:**
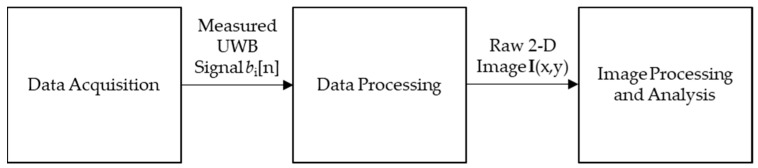
High level system block diagram for the proposed ultra-wideband imaging system.

The design of the ultra-wideband imaging system will be broken up into three main modules: a Data Acquisition module, a Data Processing module, and an Image Processing and Analysis module. Figure 1 shows the very high level block diagram for the system.

The Data Acquisition Module consists of the hardware and software needed to either simulate data or collect live data from experimental trials. Initially, the UWB device to be used for this project, the PulsON 410 (P410), was unavailable for use, so the primary source of data was from simulations in MATLAB. These simulations used the finite-difference time-domain method to provide temporal signals of UWB reflections off a simulated pot and root model.

The data from these simulation trials allowed the development of the Data Processing module without needing to wait for data from experimental trials. Additionally, it allows for the analysis of results and parameters without needing to implement time and cost intensive hardware. Processing methods such as time gating and delay-and-sum (DAS) beamforming were used to form two-dimensional (2D) images. Other methods included using Wiener filters and bandpass filters to reduce noise in the received UWB signal for the experimental trials.

The Image Processing and Analysis module measures the root depth and root diameter of the generated images. Image processing methods included modified top-hat transformations and quantization methods. Analysis tools included using energy histograms to quantify the quality of the imaging system. Both simulated results and experimental results were quantified using the same metrics to allow for the analysis of potential sources of improvement in the experimental results.

### 2.1. Simulation Data Acquisition Module Methodology

A non-contact, mono-static, single transceiver synthetic aperture radar (SAR) system using UWB frequencies was simulated to test the feasibility of this system configuration for non-destructive root imaging. This setup was selected as it provides a low-cost solution to aperture radar by requiring only one transmitter/receiver pair, and this particular set-up has been successfully applied in other fields such as hidden weapon detection [27] and breast cancer screening [28,29]. Two-dimensional simulations were done using the finite-difference time domain (FDTD) method to evaluate the feasibility of the proposed system. MATLAB was used to implement the simulations. A 2D model sufficiently modeled EM phenomena with relatively low computing power requirements (when compared to 3D FDTD simulations). Derivation and implementation of the FDTD method are found in Allen Taflove’s “Computational Electrodynamics, the finite-difference time-domain method” [30]. A configurable simulation was set up to allow for adjustable physical and electrical properties on the soil, plant roots and pot medium.

**Figure 2:**
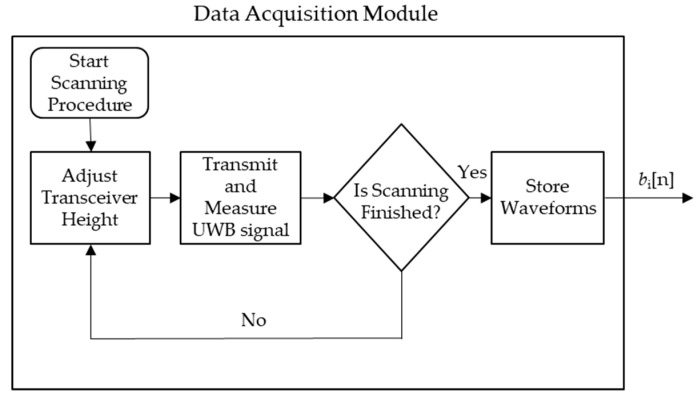
Process for the scanning procedure process for both simulated and experimental trials.

Figure 2 shows the procedure used in simulated and experimental trials to measure the UWB signals used to produce root images. The procedure produces a set of temporal scans, $b_{i}\left[n\right]$, where i=1, 2, …, N and where N is the total number of scans. $b_{1}\left[n\right]$ refers to the waveform measured when the transceiver is positioned closest to the surface of the soil, and $b_{N}\left[n\right]$ refers to the waveform measured when the transceiver is positioned closest to the bottom of soil.

#### 2.1.1. Physical Parameters

**Figure 3:**
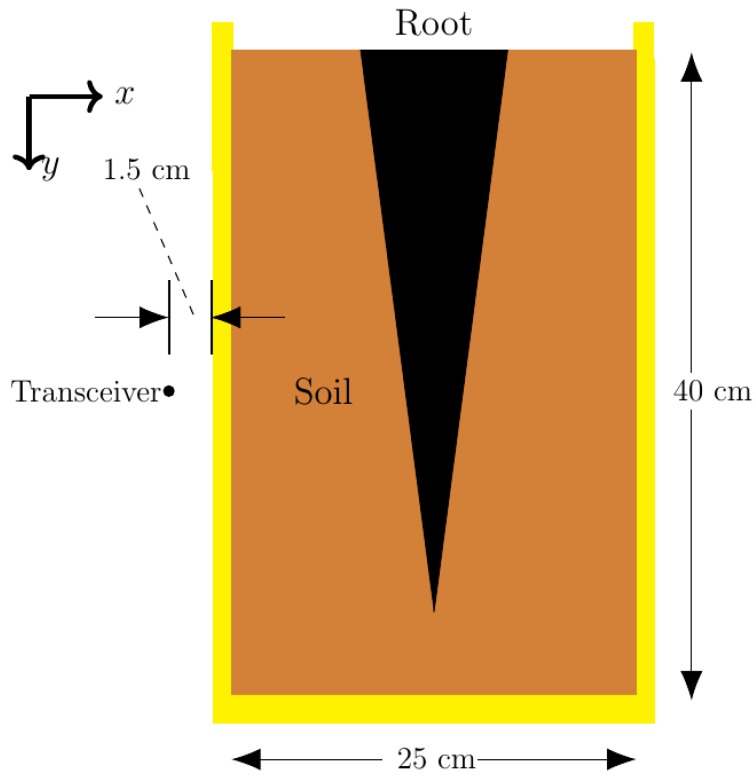
Physical parameters on pot and root model.

The simulations modeled a simple 2D potted taproot. The constant physical parameters are shown in Figure 3. The distance from the surface of the soil to the bottom interior of the pot was 40 cm while the distance from the left interior of the pot to the right interior of the pot was 25 cm. The root diameter and root depth were adjustable parameters during simulations. The pot wall width was determined to minimize reflections at the carrier frequency. The distance of the transceiver to the exterior pot wall was dependent on the thickness of the pot wall, but was approximately 1.5 cm in most simulations. The number of scans and the size of the root were varied to determine the limitations of the imaging system developed. The full results and analysis of these scans are discussed in Section 3.1, since an understanding of the Data Processing and Image Processing and Analysis Modules are needed for the discussion.

#### 2.1.2. Electrical Parameters

Accurate electrical parameters for all materials in the system being simulated are needed for good simulation results that are representative of real life phenomena. The most important electrical parameters for performing FDTD simulations are the relative permeability and relative permittivity. For the most part, the materials concerned with in this project were non-magnetic, which simplified the implementation of FDTD. The difficult parameter to determine was the relative permittivity of the materials involved. 

In particular, the soil relative permittivity at high frequencies can be modeled in many ways, each with different results [31,32,33]. The accuracy of the models was highly dependent on soil characteristics such as soil composition and moisture content [31]. A simple linear, isotropic, and non-dispersive model was used and was found to be sufficient to model experimental results. However, real time measurement and modelling of soil is a major topic of research that needs to be done to improve non-destructive root imaging techniques that rely on electromagnetic phenomena.

**Figure 4:**
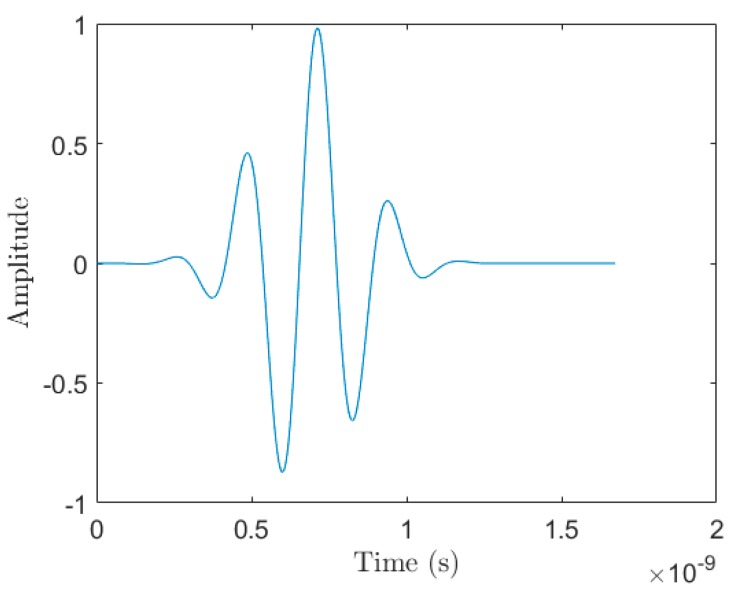
The 3.1 GHz to 5.3 GHz source waveform, g(t), used in the 2D simulations.

The Gaussian pulse waveform g(t) that is injected into the simulations as the source waveform is defined as:

(1) $$\;g\left(t\right)=exp\left(-{\left(\frac{t-3\times 2.2\;\times \;10^{9}}{2.2\;\times \;10^{9}}\right)}^{2}\right)\times \cos\left(2\pi \times 4.3\times 10^{9}\times t\right)$$

and contains most of its energy between 3.1 GHz and 5.3 GHz. Figure 4 shows the waveform, g(t), produced by Equation (1).

### 2.2. Experimental Data Acquisition Module Methodology

The UWB device used for transmitting and receiving the UWB signals is the PulsOn 410 (P410), and detailed information can be found on the Time Domain website [34]. The device transmits a 3.1 GHz–5.3 GHz pulse and relays its measurements, sampled at approximately 16.4 GSa/s, via a universal serial bus (USB) connection. The device is compact, sitting on a 7.6 × 8.0 cm board, which allows for portability and ease of use.

#### 2.2.1. Apparatus and Scanning Set-Up

**Figure 5:**
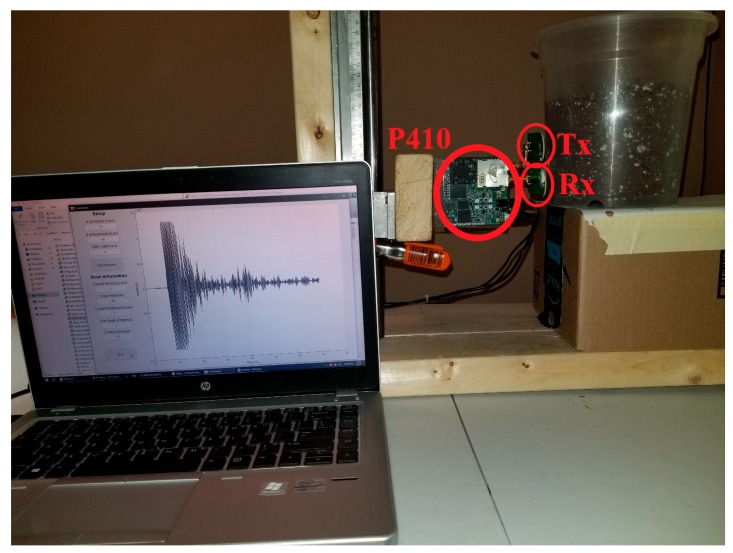
Apparatus set-up for scanning buried roots.

An apparatus was constructed to hold the P410 device allowed for easy adjustment of the transceiver height using a ball bearing platform and a clamping tool. Calibrating tests were done to measure the device’s delays. Since the P410 does not have a collocated transceiver, a non-contact, bi-static SAR scanning procedure was used. The location of the transmitter was used as reference during the scans and processing. The P410 device has the receiver and transmitter separated by 4 cm from center to center. Figure 5 shows the scanning set-up for the system.

The P410 was interfaced with MATLAB using a USB port. A graphical user interface (GUI) was designed in MATLAB to streamline the scanning process and shorten scanning times. For each buried root, four sets of vertical scans positioned at 0°, 90°, 180°, and 270° were done because this allows us to construct a cross-sectional image of the buried root to be measured for depth and average diameter. Four sets of scans provided a low scan time while providing enough images to measure important root depths and diameters. In the future, with an automated scanning system to reduce data acquisition time, a larger number of sets of scans can be made to provide more information on the buried root. Angles 0° and 180° formed cross-section side 1 and angles 90° and 270° formed cross-section side 2 on each carrot. Each vertical scan consists of 10 individual scans spaced 1 cm apart to minimize scanning times and the presence of scanning artifacts, as determined in Section 3.1.1. Similar to the simulations, the scans began at 2 cm from the surface of the soil to minimize unwanted interference from the soil-air interface at the surface of the soil.

#### 2.2.2. Pot and Root Characteristics

**Figure 6:**
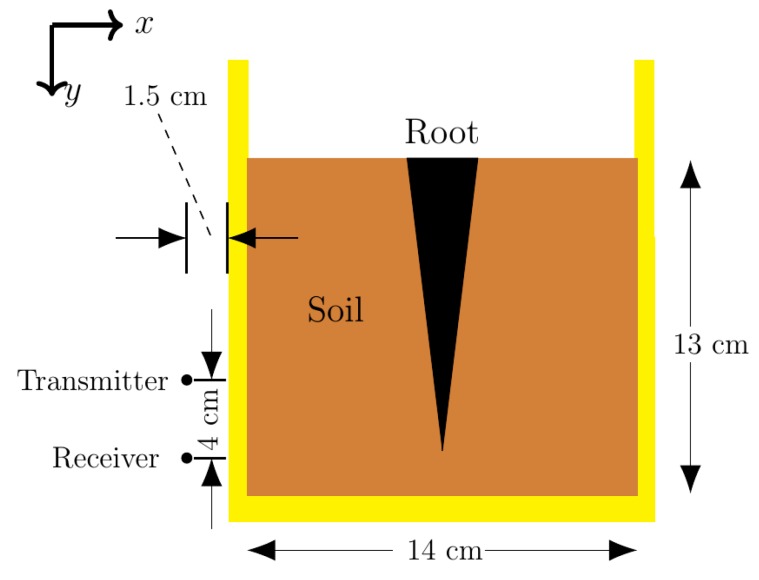
Experimental apparatus set-up dimensions for scanning buried roots.

Figure 6 shows the physical dimensions of the potted taproot. The main difference between the experimental set-up and the simulated set-up was the separation of the transmitter and receiver by 4 cm, since the P410 uses a separate antenna for transmitting and receiving. The vertical positions of the transmitter were adjusted based on the center of the transmitting antenna. Other differences included the depth of the soil, size of the pot, and size of the taproot (a carrot was chosen), due to the availability of materials for the apparatus. Two carrots were scanned, Carrot 1 with approximately 6.3 cm depth and 2.2 cm average diameter, and Carrot 2 with approximately 5.6 cm depth and 2.1 cm average diameter. These sizes were chosen to match the root sizes which were found to be problematic in simulations as found in Section 3.1.1. The scan height measurement is taken from the center of radiation of the transmitter. Additional results on other types of taproots are found in Appendix A.

#### 2.2.3. Data Acquisition Module Output Data

**Figure 7:**
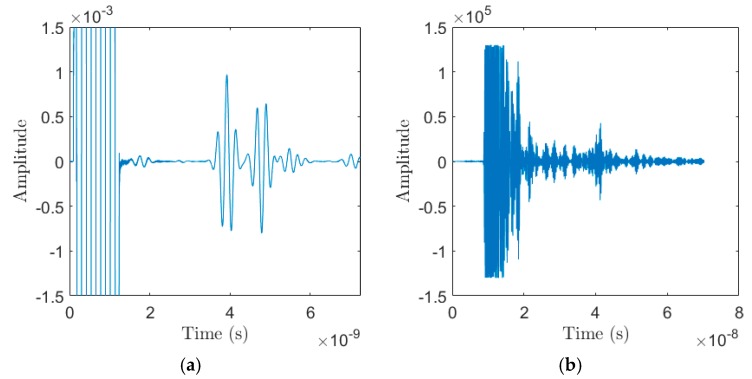
Data Acquisition Module output: (**a**) Example of a measured reflected waveform in simulated trials; (**b**) Example of a measured reflected waveform in experimental trials.

The output of the simulated and experimental trials consisted of temporal data measuring electromagnetic reflections off the system under test. The simulations resulted in a reflected wave measured as shown in Figure 7a. This waveform was for one scan at a constant vertical height. These reflections allowed us to generate an energy mapping of the system under test showing where the most reflective materials were within the soil. These reflections were the primary output of the Data Acquisition Module. Note that Figure 7a has the y-axis scaled to show the amplitude of the reflections at 3 ns to 7 ns. The high amplitude measurements before 2 ns were the measurements of the source waveform. Figure 7b shows an example of a reflected waveform measured by the P410 in the experimental trials. The P410 was originally meant for long distance ranging purposes, so at a minimum it collects 70 ns of measurements. 

With the selected physical parameters, the region of interest lay at around the 12 ns–13 ns for calculating the DAS beamforming image. Unfortunately, this portion was in an area with large amounts of noise caused by the hardware in the P410 [34]. This hardware noise was dealt with by using the procedure outlined in Section 2.3.1.

As previously defined, $b_{i}\left[n\right]$ is the set of measured reflected waveforms, where i=1, 2, …, N and where N is the total number of scans. $b_{1}\left[n\right]$ refers to the waveform measured when the transceiver is positioned closest to the surface of the soil and $b_{N}\left[n\right]$ refers to the waveform measured when the transceiver is positioned closest to the bottom of soil.

### 2.3. Data Processing Module Methodology

**Figure 8:**
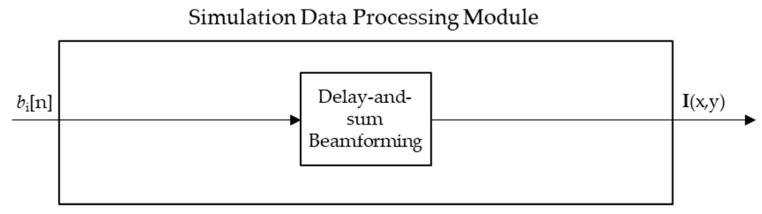
Data Processing Module blocks for the simulated trials.

**Figure 9:**
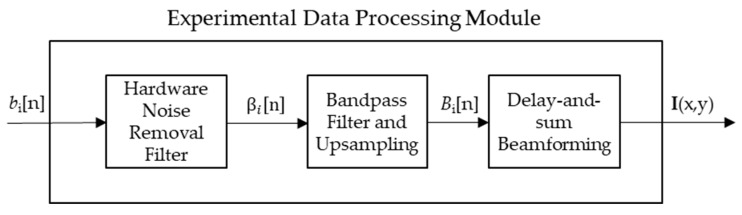
Data Processing Module blocks for the experimental trials.

The Data Acquisition Module produces a set of reflected waveforms $b_{i}\left(n\right)$. The peak energy occurrences in each waveform $b_{i}\left(n\right)\;$ correlate to approximate distances from transceiver to reflecting material. Delay-and-sum (DAS) beamforming forms an image based on known physical and electrical parameters using $b_{i}\left(n\right)$, and is the primary data processing method for creating a 2D spatial image using 1D temporal data. DAS was chosen over other beamforming algorithms due to its success in other imaging studies utilizing UWB systems [26,28,29]. The simulated results were designed to be absent of noise and required no preprocessing before the delay-and-sum beamforming algorithm, as shown in Figure 8. For the experimental trials, the collected data required some pre-processing to remove noise caused by the apparatus as shown in Figure 9. The details of the additional noise removal is outlined in Section 2.3.1. The details of the DAS beamforming algorithm is outlined in Section 2.3.2.

#### 2.3.1. Experimental Trial Noise Removal

The development of the Wiener filter uses Section 18.6.3 of online notes provided by S.C. Douglas as reference [35].

**Figure 10:**
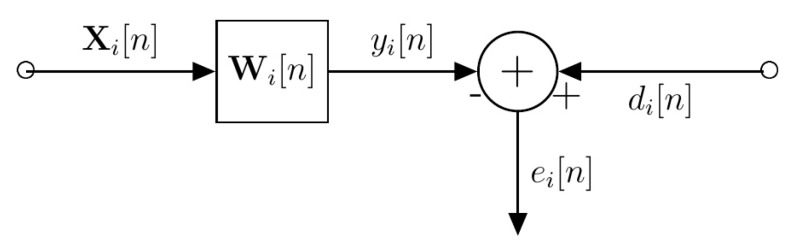
Block diagram for hardware noise removal system.

As seen in Figure 6b, there was significant noise in the measured signals caused by the hardware in the first 15 ns of the captured waveform [34]. Since the noise was consistent in terms of amplitude and temporal location, the Wiener filter estimated the noise on the assumption that the rest of the signal consisted of only additive random processes. Figure 10 shows the general block diagram for the process to remove the hardware noise.

The goal of a Wiener filter is to design the filter, W[n], to minimize the mean-squared error cost function, $J_{\mathrm{MSE}}\left[n\right]$. $J_{\mathrm{MSE}}\left[n\right]$ is defined as:

(2) $$\;J_{\mathrm{MSE}}\left[n\right]=\frac{1}{2}E\left(e^{2}\left[n\right]\right)\;$$

where $E\left(e^{2}\left[n\right]\right)$ is the mean squared error. Given $b_{i}\left[n\right]$ where i=1,2,…, N scans and Equation (2), we define the estimated hardware noise for the *i*th scan to be $y_{i}\left[n\right]$. We let the desired signal be $d_{i}\left[n\right]=b_{i}\left[n\right]$. The input $X_{i}\left[n\right]$ is a matrix which contains all the scans except for the *i*th scan. The optimal values for $W_{i}\left[n\right]$ that minimize $J_{i,\mathrm{MSE}}\left[n\right]$ for the *i*th scan is:

(3) $$\;W_{i}\left[n\right]=E{\left(X_{i}\left[n\right]X_{i}^{T}\left[n\right]\right)}^{-1}\left[n\right]E\left(d_{i}\left[n\right]X_{i}\left[n\right]\right)\;$$

Equation (3) calculates the required filter coefficients to minimize $J_{i,\mathrm{MSE}}\left[n\right]$. We can now calculate $y_{i}\left[n\right]$, which is the estimated hardware noise for the *i*th scan using:

(4) $$\;y_{i}\left[n\right]=\;W_{i}\left[n\right]X_{i}\left[n\right]\;$$

which is used to calculate the measured reflected signal with noise removed:

(5) $$\;\beta_{i}\left[n\right]=b_{i}\left[n\right]-y_{i}\left[n\right]\;$$

**Figure 11:**
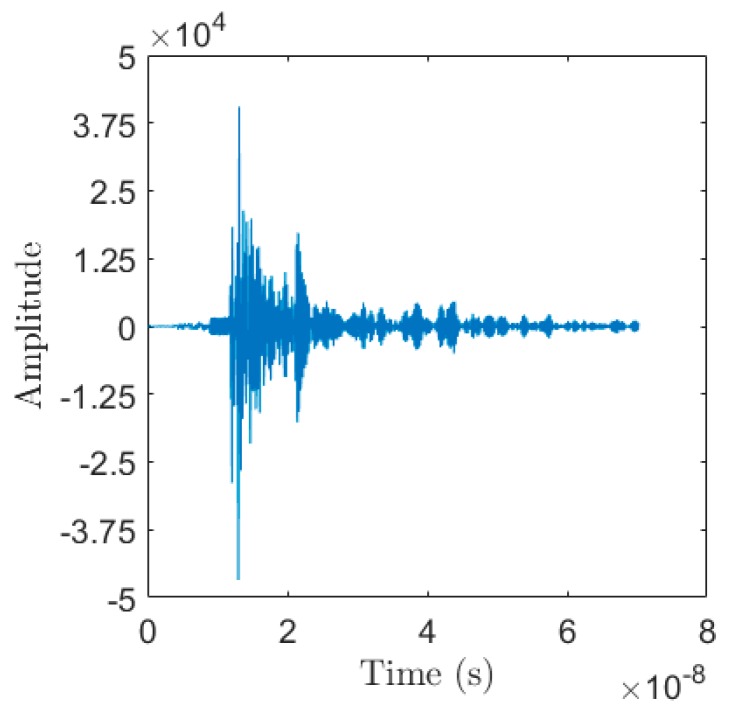
The measured reflected signal with the hardware noise removed, $\beta_{i}\left[n\right]$.

Figure 11 shows the $\beta_{i}\left[n\right]$, the measured reflected signal with the hardware noise removed.

Next, a bandpass filter with cut-off frequencies of 3.1 GHz to 5.3 GHz was used to remove unwanted electromagnetic noise at frequencies beyond the frequency of the P410. The Canadian Table of Frequency Allocations specifies many uses for frequencies in and around the P410 device for mobile use [36]. In particular, Wi-Fi and Bluetooth services operate around 2.4 GHz and is present in the spectrum of each Wiener filtered scan $\beta_{i}\left[n\right]$. The filter was designed with MATLAB’s *designfilt* command to be a bandpass filter with order 100 [37]. The order was chosen to be very large to ensure adequate attenuation outside the pass band.

There are many factors which need to be considered in the resolution capabilities of this system, with some potential issues (which arise due to the delay-and-sum beamforming method) regarding the sampling rate of the P410 is eliminated by upsampling and interpolating. MATLAB’s *interp* function upsamples a given signal and then interpolates the new samples to minimize the mean-square error between the new samples and their ideal values [37]. $B_{i}\left[n\right]$ will be used to denote the final filtered, upsampled, and interpolated signal. An upsampling factor of 10 (which decreases sampling period to 6.1 ps from 61 ps) was determined using root measurements to have the best results on average for all scans taken.

#### 2.3.2. Delay-and-Sum Beamforming

The formulation of the DAS beamforming algorithm in this section used Chapter 4.10 in the Handbook of Ultra-Wideband Short-Range Sensing Theory, Sensors, Applications as a reference [26]. Modifications were made to optimize the algorithm for maximizing the quality of the imaged potted roots.

A steering vector $h_{i}^{\left(x_{r},y_{r}\right)}\left(t\right)$ needs to be defined and it is an impulse function with a time delay equal to the time it takes a wave to travel the most direct path from transceiver at the *i*th scanning position to imaging point $\left(x_{r},y_{r}\right)$ and back to the transceiver. The distance that the wave traveled is needed to calculate the appropriate time delay for $h_{i}^{\left(x_{r},y_{r}\right)}\left(t\right)$.

**Figure 12:**
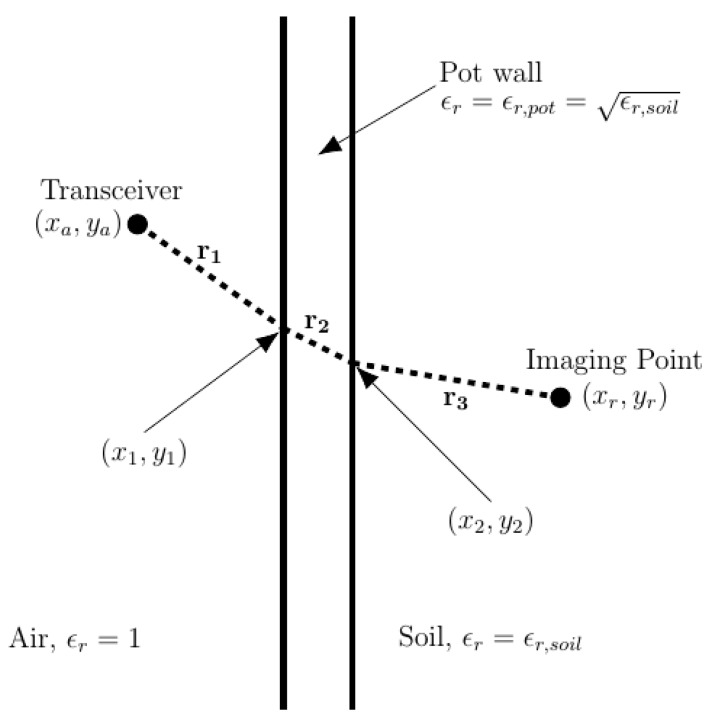
Most direct path from transceiver to imaging point for use in delay-and-sum (DAS) beamforming.

Figure 12 shows the vectors $r_{1}$, $r_{2,}$ and $r_{3}$ that determines the most direct path that a wave can travel from transceiver to imaging point. The values $x_{a},y_{a},x_{1},x_{2},x_{r},y_{r},\epsilon_{r,pot},\epsilon_{r,soil}$ are known, and $y_{1},y_{2}$ are not known. To determine the magnitudes of the vectors $r_{1}$, $r_{2}$, and $r_{3}$, we must first find $y_{1}$ and $y_{2}$. Using $n=\sqrt{\left(\mu_{r}\epsilon_{r}\right)}$ and Snell’s law, we can solve for $y_{1}$ and $y_{2}$ using the following equations:

(6) $$\;\frac{\left|y_{1}-y_{a}\right|}{\sqrt{{\left(x_{1}-x_{a}\right)}^{2}+{\left(y_{1}-y_{a}\right)}^{2}}}\sqrt{\epsilon_{r,air}}=\frac{\left|y_{2}-y_{1}\right|}{\sqrt{{\left(x_{2}-x_{1}\right)}^{2}+{\left(y_{2}-y_{1}\right)}^{2}}}\sqrt{\epsilon_{r,pot}}\;$$

(7) $$\;\frac{\left|y_{2}-y_{1}\right|}{\sqrt{{\left(x_{2}-x_{1}\right)}^{2}+{\left(y_{2}-y_{1}\right)}^{2}}}\sqrt{\epsilon_{r,pot}}=\frac{\left|y_{r}-y_{2}\right|}{\sqrt{{\left(x_{r}-x_{2}\right)}^{2}+{\left(y_{r}-y_{2}\right)}^{2}}}\sqrt{\epsilon_{r,soil}}\;$$

Equations (6) and (7) are difficult to solve analytically so MATLAB’s *fzero* function is used to solve for $y_{1}$ and $y_{2}$. The *fzero* function uses a combination of bisection, secant, and inverse quadratic interpolation methods to find the root of the input equations [37].

With known values for $y_{1}$ and $y_{2}$, numerical values are then calculated for $r_{1}$, $r_{2}$, and $r_{3}$. The total time of flight T can then be calculated as:

(8) $$\;T=2\left(\frac{\left|r_{1}\right|}{c_{0}}+\frac{\left|r_{2}\right|}{c_{0}}\sqrt{\epsilon_{r,pot}}+\frac{\left|r_{3}\right|}{c_{0}}\sqrt{\epsilon_{r,soil}}\right)\;$$

Note that Equation (8) has a factor of 2 to account for the entire distance from transceiver, to imaging point, back to transceiver. The steering vector is then defined to be:

(9) $$\;h_{i}^{\left(x_{r},y_{r}\right)}\left(t\right)=\delta \left(t+t_{0}+T\right)\;$$

Equation (9) is referred to as the steering vector and is essential to the performance of DAS beamforming. In application, $h_{i}^{\left(x_{r},y_{r}\right)}\left(t\right)\;$ is sampled appropriately to be $h_{i}^{\left(x_{r},y_{r}\right)}\left[n\right]$. It is important to keep in mind that for every position $\left(x_{r},y_{r}\right)$ to be imaged, there are N transceiver locations, each with their own steering vector for position $\left(x_{r},y_{r}\right)$. A series of calibration tests to determine the startup time of the P410 device to be $t_{0}\approx 11$ ns for the experimental trials. For the simulated trials, $t_{0}$ will vary dependent on the source waveform used and is generally the time to the maximum value of the waveform.

**Figure 13:**
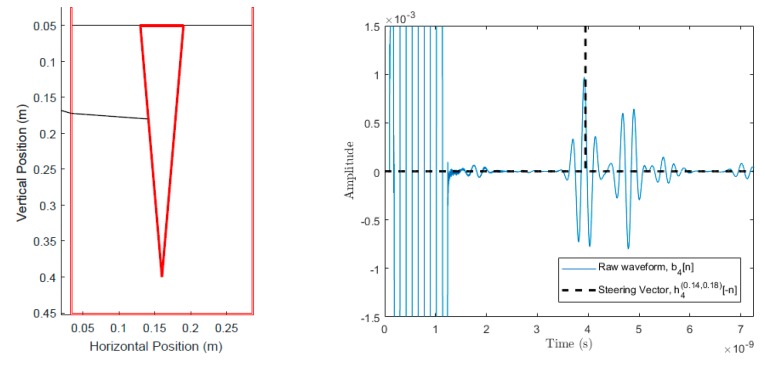
Scan position 4 and calculated ray path for the emitted ultra-wideband (UWB) pulse to point (0.14, 0.18).

A single transceiver waveform from the simulated trials will be used for the purposes of explaining the calculation of pixel intensity. Figure 13 shows the selected wave path for the example. The set of scans used for this example has 12 scans, 35 cm depth, 3 cm average diameter, 20 soil relative permittivity, and 10 root relative permittivity.

In particular, we need to first determine the steering vector for $b_{4}\left[n\right]$ at the imaging point $\left(x_{r},y_{r}\right)=\left(0.14,0.18\right)$, i.e., we are calculating $h_{4}^{\left(0.14,0.18\right)}\left[n\right]$. We can overlay the steering vector with the waveform by flipping the steering vector about n = 0 and plotting both. Figure 13 shows that the calculated steering vector lies within one of the measured reflections. This is logical because Figure 13 shows that the chosen imaging point, (0.14,0.18), is very close to the surface of the root which is causing high energy reflections.

We now define $z_{4}^{\left(0.14,0.18\right)}\left[n\right]$ to be the convolution of $h_{4}^{\left(0.14,0.18\right)}\left[n\right]$ and $b_{4}\left[n\right]$. More generally, this equation is written as:

(10) $$\;z_{i}^{\left(x_{r},y_{r}\right)}\left[n\right]=h_{i}^{\left(x_{r},y_{r}\right)}\left[n\right]*b_{i}\left[n\right]\;$$

$z_{4}^{\left(0.14,0.18\right)}\left[n\right]$ is a waveform which contains the marked portion of $b_{4}\left[n\right]$ in Figure 13 centered at n=0. The energy for the signal is then calculated using:

(11) $$\;I_{i}\left(x_{r},y_{r}\right)=D_{i}\left(x_{r},y_{r}\right)\overset{S_{n}/2}{\underset{-S_{n}/2}{\sum }}{\left(z_{i}^{\left(x_{r},y_{r}\right)}\right)}^{2}\;$$

where $I_{i}\left(x_{r},y_{r}\right)$ is the energy contribution from the *i*th scan at imaging point, $\left(x_{r},y_{r}\right)$, $D_{i}\left(x_{r},y_{r}\right)$ is a scalar weighting for the *i*th scan at imaging point $\left(x_{r},y_{r}\right)$, and $S_{n}$ is the window size of time of energy to be used for the image.

The weighting value $D_{i}\left(x_{r},y_{r}\right)$ is a scalar value from 0 to 1. The value is determined by how close the position of the transceiver is to the imaging point. The closer the position, the closer the value of $D_{i}\left(x_{r},y_{r}\right)$ is to 1 for that scan number and imaging point. This places emphasis on the scans closer to the imaging point to reduce the effect of potential multipath issues. Emphasis is placed on the positions with the shortest direct wave paths to the imaging point. Equation (12) is the equation used for determining all the values for $D_{i}\left(x_{r},y_{r}\right)$.

(12) $$\;D_{i}\left(x_{r},y_{r}\right)=1-\left|\frac{y_{i}-y_{r}}{\max\left(y_{1,2,\ldots ,N}-y_{r}\right)}\right|\;$$

where $y_{i}$ is the vertical position of the *i*th scanning position and $y_{1,2,\ldots ,N}$ is the vector containing all the scanning position’s vertical coordinates.

Since we are dealing with a finite length pulse, we choose a window size of approximately 0.23 ns, which corresponds to approximately one period of the 4.2 GHz carrier frequency (this is the same for both simulated and experimental trials). The length of $S_{n}$ is a very important parameter and analysis of adjustment of $S_{n}$ is done in Section 3.1.3. $S_{n}$ is referred to as the window size.

Equation (11) for i = 4 and $\left(x_{r},y_{r}\right)=\left(0.14,0.18\right)$ evaluates to $7.34\times 10^{-9}$. This value is the energy contribution of the 4th scan for the pixel at (0.14,0.18).

The process consisting of calculating is repeated for all N scan positions. The pixel intensity for imaging point $\left(x_{r},y_{r}\right)$ for the final image is given by summing all the contributions of each scan:

(13) $$\;I\left(x_{r},y_{r}\right)=\overset{N}{\underset{i=1}{\sum }}I_{i}\left(x_{r},y_{r}\right)\;$$

If $x_{r}$ and $y_{r}$ in Equation (13) are changed to the vectors $x_{r}$ and $y_{r}$ corresponding to a spatial grid in the Cartesian coordinate system, then $I\left(x_{r},y_{r}\right)$ is rewritten as a matrix I(x,y) and is the unprocessed DAS beamforming image for this particular set of scans. Careful design of vectors $x_{r}$ and $y_{r}$ is done to exploit known knowledge of the root to reduce noise in the image and to reduce the time it takes to create an image. For example, if it is physically observed that the stem of a canola plant right at the surface of the soil exists 3 cm from the pot, then it is likely that the root should be around 3 cm from the pot. $x_{r}$ and $y_{r}$ can be selected so that it images the region around 3 cm from the pot wall in this case, ignoring regions that may be too far from the stem location of the plant.

#### 2.3.3. Data Processing Module Output Data

**Figure 14:**
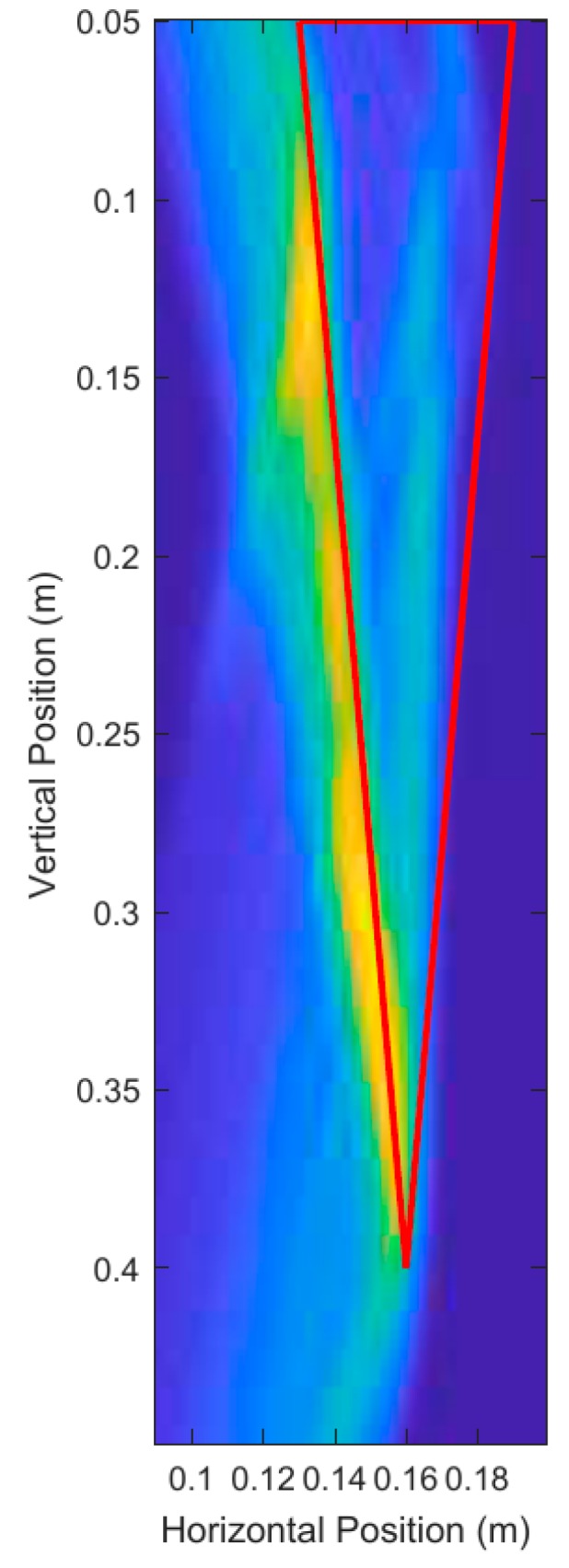
Scan position 4 and calculated ray path for emitted UWB pulse to point (0.14, 0.18). The thick red lines indicate the true position of the root and are not a part of the output of the system.

The primary purpose of the Data Processing Module is to produce a spatial 2-D image as defined by I(x,y). The image should contain enough information on the energy distribution of the contents of the pot to be able to measure the surface location of the root. Figure 14 shows the result of the image calculated using DAS beamforming. Note that the thick line outlining the root is the true location of the root, and is not a part of I(x,y).

I(x,y) is used in the Image Processing and Analysis Module to improve the quality of the image and measure important root characteristics such as depth and average diameter.

### 2.4. Image Processing and Analysis Module Methodology

**Figure 15:**
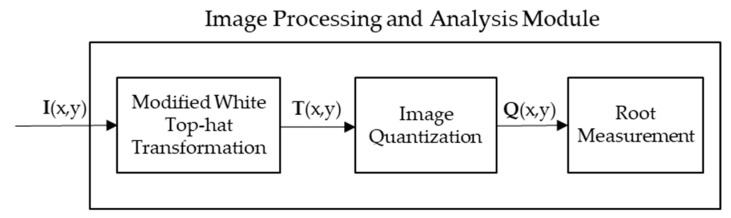
Image Processing and Analysis Module blocks for simulated and experimental trials.

This section covers the image processing methods used to isolate the location of the root surface in the images produced by the Data Processing module. It is important to note that these methods are created assuming that the true location and size of the root is unknown. Creating this module in an unsupervised fashion will help in adapting the system to the experimental trials where information on a live root is unavailable. This section makes heavy use of MATLAB’s Image Processing Toolbox [37]. Figure 15 shows the flow of the Image Processing and Analysis Module blocks that will be described in this subsection. 

#### 2.4.1. Morphological Transformations

The primary image processing technique used to improve the image quality of the unprocessed DAS beamforming images is a modified white top-hat transform. A normal white top-hat transform takes a structuring element and performs a series of morphological transformations with the element to the desired image. The modified white top-hat transform does a similar series of morphological transformations. A structuring element is a 2D vector containing a shape that determines the size of details to retain. Equation (14) shows the operations needed to perform the modified white top-hat transformation.

(14)  T(x,y)=I(x,y)+((I(x,y)⊖b)⊕c) 

First, an erosion on I(x,y) is performed, which removes bright artifacts that are smaller than the chosen size of b. Consequently, this also removes the edges of larger high intensity regions. A dilation is performed using c to restore the edges of the larger high intensity regions. The result of ((I(x,y)⊖b)⊕c) is an image with small artifacts removed and larger high intensity regions emphasized. The final image T(x,y) becomes an image with the larger, high intensity regions emphasized over smaller, lower intensity regions. The variation of the optimal design of structural elements b and c is dependent on the characteristics of I(x,y), and they are critical to the performance of the analysis module and are analyzed in Section 3.1.4.

#### 2.4.2. Energy Histograms, Image Quantization, Erosion, and Interpolation

An important used for analyzing and improving the quality of I(x,y) are the energy histograms. In particular, the energy histogram allows us to analyze the energy distribution of the image and remove unnecessary information.

The development of energy histograms starts with more conventional image histograms. First, I(x,y) is normalized to contain values ranging from 0–255, which is standard for 8-bit grayscale images. It is important to note that the values are not rounded to prevent loss in precision during processing and analysis. The image histogram of I(x,y) divides the pixel values into bins from 0–255 and places all the pixels in the image into the bin corresponding to each pixel’s intensity.

The energy contribution of each bin is calculated by multiplying the bin number with the number of pixels in that bin. Plotting the energy contribution of each bin against the bin number (pixel intensity) will be defined as the energy histogram, and the energy histogram effectively maps how much energy is present at individual pixels relative to the energy in the entire image. Integrating the energy histogram and redefining the *y*-axis to be a percentage of the total energy creates a cumulative energy histogram.

**Figure 16:**
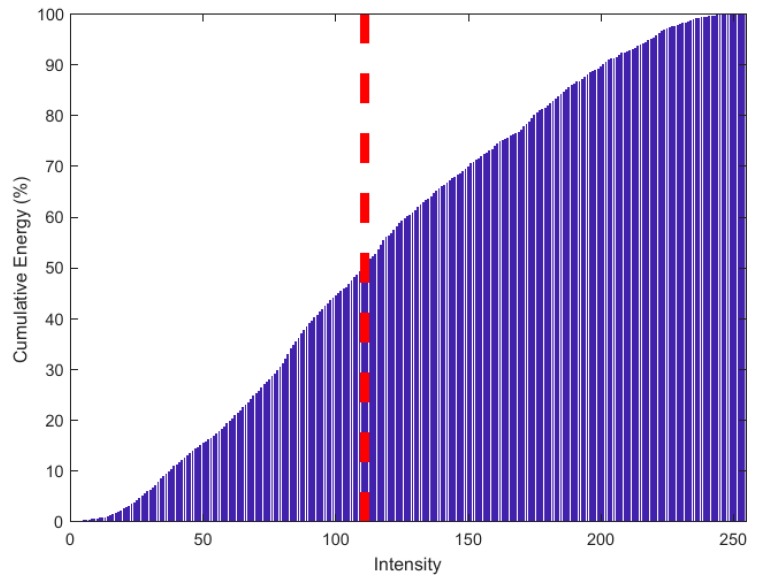
Cumulative energy histogram for I(x,y) shown in Figure 14. The red dashed line indicates the 50% energy threshold.

The cumulative energy histogram of T(x,y) shown in Figure 16 was analyzed and a binary mask was made by retaining only the pixel intensities above a specified threshold intensity, which was related to a percentage of energy to retain in the image. For example, say 50% of the energy in the image is contained in bin numbers (pixel intensities) 111–255 and we want to keep that 50%. All pixels in the range 111–255 are retained as a binary ‘1’, and all other pixels not in this range are retained as a binary ‘0’. This is done to analyze and process the potential location of the root based on size and shape alone. Creating a binary mask helps to remove high energy noise which may compete with the energy of the reflections off the surface of the root.

The threshold intensity is an essential parameter that is dependent on the characteristics of T(x,y) and is analyzed in Section 3.1.5. A binary erosion transformation is done to remove unwanted smaller artifacts at the edges of the root surface. The largest area has half of its pixels removed, starting from the side closest to the pot to the center of the binary area. This is done to find the center of the largest area which should correspond to the location of the root. The remaining image, denoted is then interpolated to the center of the pot and then defined as Q(x,y). Cross sections of a root are formed by concatenating images of scans taken on opposing sides of the diameter of the pot. The height of the cross section is measured by collapsing the columns with a bitwise ‘or’ operation and then summing the remaining pixels. The average diameter is calculated by dividing the measured area by the measured height.

## 3. Results

This section will cover the results from the simulated trials, the experimental trials, and replication of the experimental trials in simulation.

### 3.1. Simulated Trial Results

This subsection covers the results and their interpretations for the simulated trials covering the physical parameters, electrical parameters, data processing parameters, and image processing parameters of the proposed UWB root imaging system. The simulated trials used the methodology outlined in Section 2.1, Section 2.3 and Section 2.4.

#### 3.1.1. Physical Parameter Adjustment Results

**Figure 17:**
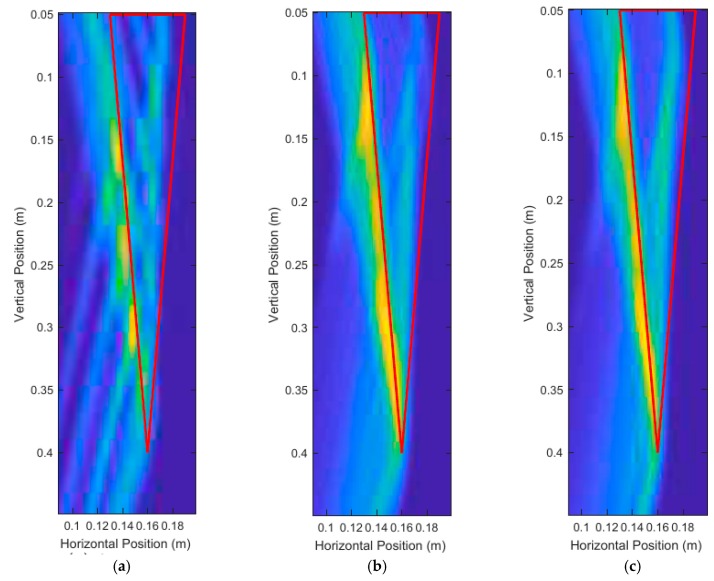
Unprocessed DAS beamforming images for: (**a**) six scanning positions, (**b**) 12 scanning positions, and (**c**) 21 scanning positions.

A large taproot with a 35 cm depth and 3 cm average diameter was scanned. The size of this taproot was approximately the size of a fully grown canola root [38]. The vertical scanning position began at a depth of 2 cm from the surface and ended at 41 cm (which was 2 cm from the bottom of the pot). A total of 21, 12, and 6 equally spaced scans were used, and the unprocessed DAS beamforming results are shown in Figure 17. Each scan consisted of running the simulations and injecting the 3.1 GHz–5.3 GHz source waveform at the current transceiver location, and subsequently measuring the reflected energy at the transceiver location. These simulations were run with a high permittivity contrast between soil ($\epsilon_{r,soil}=20$) and root ($\epsilon_{r,root}=10$) and no noise to maximize desired reflected energy. The soil relative permittivity was selected to be 20, to match literature on dry soil relative permittivity at around 4 GHz [32,39]. The measurement results are summarized on Table 1. These measurements were made by keeping 35% of the energy for all scans, and a structuring element of size 2 cm diameter for the modified top-hat transformations, and 1 mm diameter for the binary mask erosion.

The thick red lines in Figure 17 indicate the true position of the root and are not a part of the output of the system. Figure 17a shows that six scans produced very distinct artifacts that were not present in Figure 17b,c. Judging qualitatively from the results in Figure 17, 12 scans performed quite similarly to 21 scans while taking effectively half the time to simulate and process. Table 1 confirms the qualitative observations with quantitative measurements, with 12 scans and 21 scans performing very similarly in terms of error. It is important to note that it is not the number of scans that is important, but the size of the spacing between the scans. In this case, we were dealing with a constant vertical scanning range so the spacing was only a function of the number of scans.

Next, with 12 scans per root, the size of the root was varied to determine how the root size would affect the imaging results. Table 2 summarizes the sizes simulated and the errors in measurements. These measurements were made by keeping 35% of the energy for all scans and a structuring element of size 2 cm diameter for the modified top-hat transformation, and 2 mm diameter for the binary mask erosion.

**Figure 18:**
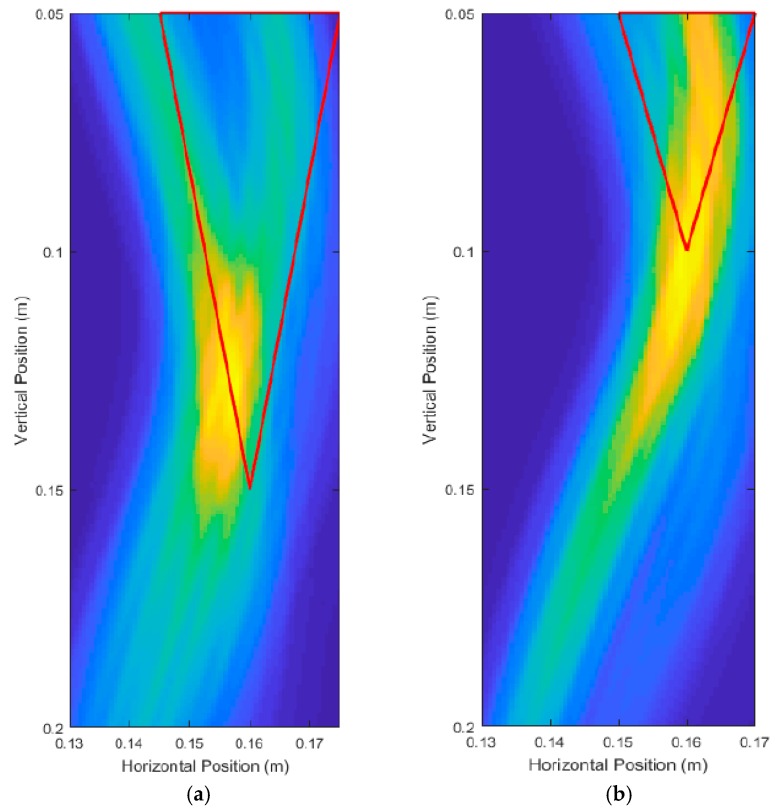
Unprocessed DAS beamforming images for a root with: (**a**) 10.00 cm depth and 1.50 cm average diameter, and (**b**) 5.00 cm depth and 1.00 cm average diameter.

Table 2 shows increasing errors as the root sizes became smaller. In particular, the roots with 10.00 cm depth and 5.00 cm depth had very large percent errors in depth measurement, with 30.4% and 112.8%, respectively. Examining the unprocessed images in Figure 18 shows that there was a large amount of energy from that back side of the root (the side furthest from the transceiver), causing wave interference with the front side reflections, and erroneous detection of the front side location. The unsupervised data and image processing methods used for measurements were unable to determine the true location of the front surface of the root, causing large measurement errors which made the measured depth and diameter unreliable metrics, and thus these were omitted.

**Figure 19:**
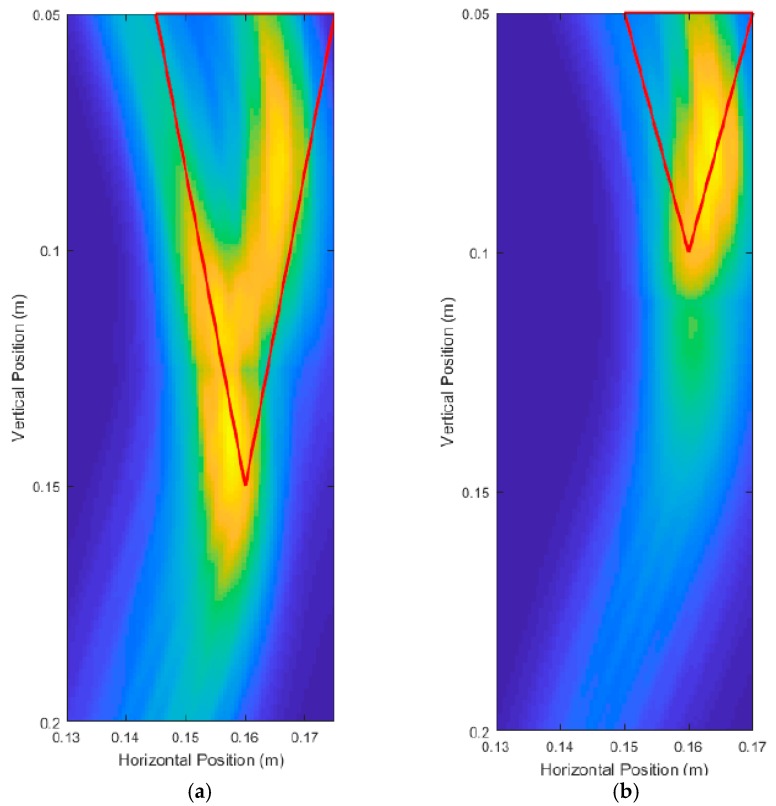
Unprocessed DAS beamforming images with a shortened scan range (decreased scan spacing) for a root with: (**a**) 10.00 cm depth and 1.50 cm average diameter, and (**b**) 5.00 cm depth and 1.00 cm average diameter.

In efforts to improve the unprocessed image quality, the scanning area was reduced from a range of 2 cm to 41 cm. For Root Test Number 3, the range became 2 cm to 17 cm. For Root Test number 4, the range became 2 cm to 10 cm. This effectively decreased the scan spacing from 3.36 cm to 1.33 cm for Root Test Number 3, and 3.36 cm to 0.75 cm for Root Test Number 4. Figure 19 shows the unprocessed images produced and showed some improvement in the quality of the image. The surface of the root was still poorly detected, but the energy was better concentrated around the location of the root. Figure 18 and Figure 19 root sizes show the limitation of the current UWB imaging system for imaging smaller roots, with slight improvements possible through using decreased scan spacings.

#### 3.1.2. Electrical Parameter Adjustment Results

Twelve equally spaced scans were done on a 35 cm deep root with an average diameter of 3 cm. Macroscopic scattering objects were added to the soil each with randomly generated sizes and permittivities to demonstrate the importance of having a high root to soil relative permittivity contrast. We kept the soil relative permittivity at 20, $\epsilon_{r,soil}=20$, again to match literature for dry soils at around 4 GHz [32,39]. 

**Figure 20:**
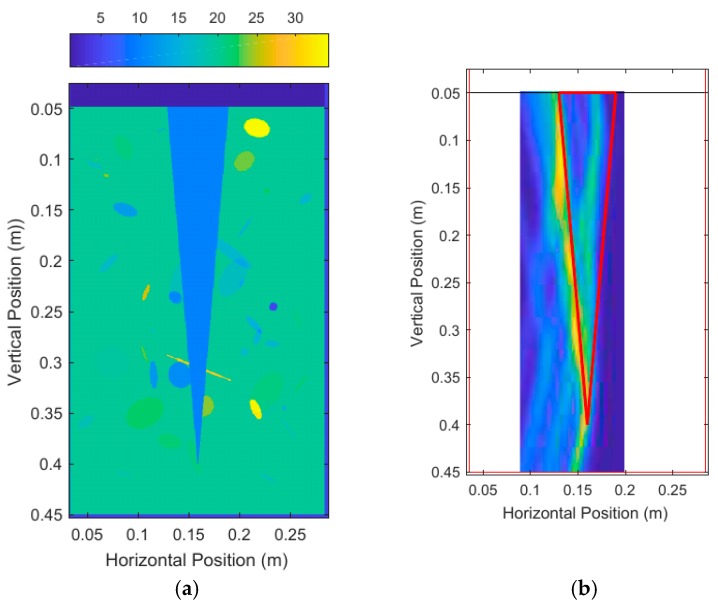
The noisy system under test containing a root with $\epsilon_{r,soil}=10$ where: (**a**) is the relative permittivity distribution of the simulated system and (**b**) is the unprocessed DAS beamforming image for the simulated system.

Figure 20 shows that the introduction of macroscopic scattering objects slightly degraded the quality of the image when compared to Figure 17b, which was imaged using the same simulation parameters but without the macroscopic scattering objects. There were more artifacts around the surface of the root as well as at the bottom tip of the root in Figure 20b. The overall quality was still quite good as the majority of the energy in the image was concentrated around the front surface of the root.

**Figure 21:**
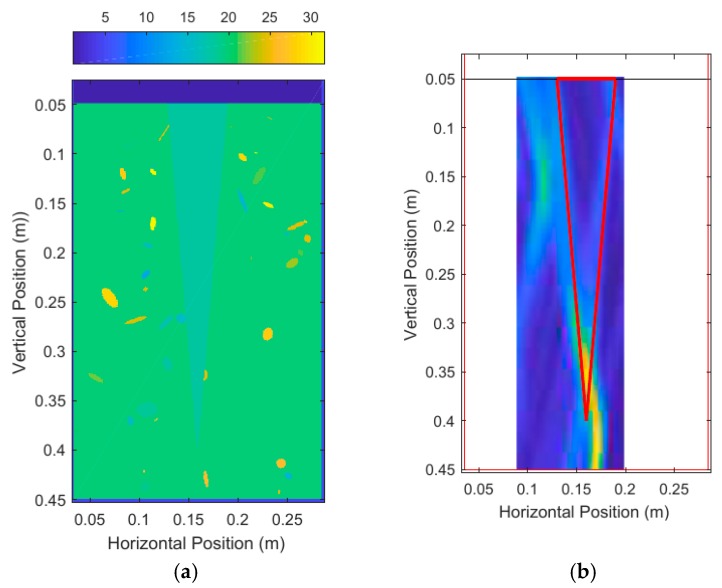
The noisy system under test containing a root with $\epsilon_{r,soil}=18$ where: (**a**) is the relative permittivity distribution of the simulated system and (**b**) is the unprocessed DAS beamforming image for the simulated system.

For the root with $\epsilon_{r,root}=18$, the relative permittivity contrast between the macroscopic scatterers and soil was large when compared to the relative permittivity contrast between root and soil. The low root-to-soil relative permittivity contrast caused low energy reflections which became distorted by the high energy reflections off the scatterers. Figure 21 shows the image quality was severely reduced. Most of the energy in the image was concentrated around an area that was not near the front surface of the root. It would be very difficult to determine which regions of the image was related to the root without prior knowledge of the location of the root. Figure 21 demonstrates the limitations of the UWB imaging system designed when operating under noisy conditions with low root-to-soil relative permittivity contrast. It becomes impossible to separate information on the root from unwanted noise with a low relative permittivity contrast using this system alone. This has been a common issue for other non-destructive root imaging methods such as MRI [13] and GPR [40].

#### 3.1.3. Window Size Parameter Adjustment Results

**Figure 22:**
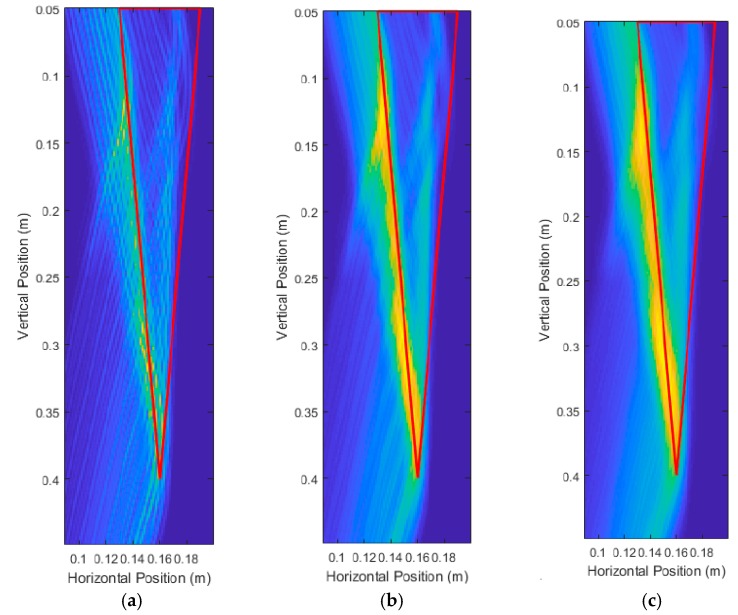
Unprocessed DAS beamforming images where $S_{n}$ is set to be: (**a**) quarter carrier cycle length, (**b**) half carrier cycle length, and (**c**) one-and-half carrier cycle length.

The window size $S_{n}$ selected for the DAS beamforming algorithm needs to be carefully determined based on the period of the carrier frequency. If the window size is too short, then the energy contributions due to the oscillations of the carrier frequency become apparent. Figure 22a uses a quarter cycle length for the window size and has artifacts caused by the carrier frequency oscillations. Figure 22b uses a half cycle for the window size and contains some artifacts caused by the carrier frequency. Figure 22c uses a one-and-half cycle length for the window size and there are no artifacts caused by the carrier frequency. However, too large of a window size puts the DAS beamformed image at a higher risk of having artifacts caused by signal interference due to the non-zero length of the UWB pulses. Larger window sizes will also have larger regions of high intensity, which can be seen in Figure 22c when compared with Figure 17b, as these figures had identical parameters other than the window size. As such, around one cycle length is optimal for the beamforming algorithm, long enough to avoid carrier frequency artifacts and short enough to minimize reflection interference. All images produced for this paper used a one cycle-length window size unless otherwise stated.

#### 3.1.4. Morphological Transformation Parameter Adjustment Results

**Figure 23:**
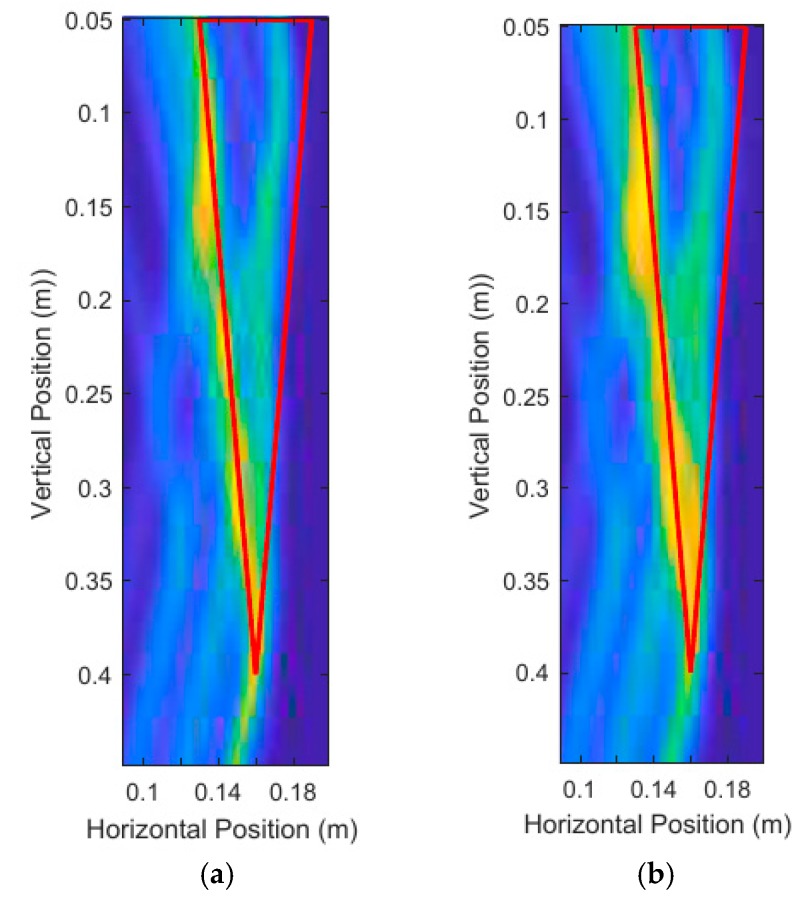
DAS beamforming results for: (**a**) the unprocessed image and (**b**) the image after the modified top-hat transformation.

The modified top-hat transformation, defined by Equation (14) in Section 2.4.1, was useful for emphasizing the surface of the roots in the DAS beamforming images. The transformation raised the intensity of larger objects in the image (which is generally the root) while reducing the intensity of smaller objects. The optimal selection of b and c is dependent on the characteristics of the root to be imaged. Generally, c will be selected to be roughly the same size and shape as b to restore any lost information on the surface of interest. The optimal size and shape for b is dependent on how large the root being imaged is. The larger the root, the larger b can be, in order to reduce the intensity of smaller artifacts present in the image. Figure 23 compares the unprocessed image and the processed image on a root that has been distorted by macroscopic objects. A circular structuring element approximately 4 mm in diameter is used for both b and c.

The areas associated with the surface of the root become emphasized, while some distortions, such as the high intensity distortion near the bottom tip of the root, are de-emphasized. Overall, this helps the unsupervised image processing method to identify the most likely location of the root.

**Figure 24:**
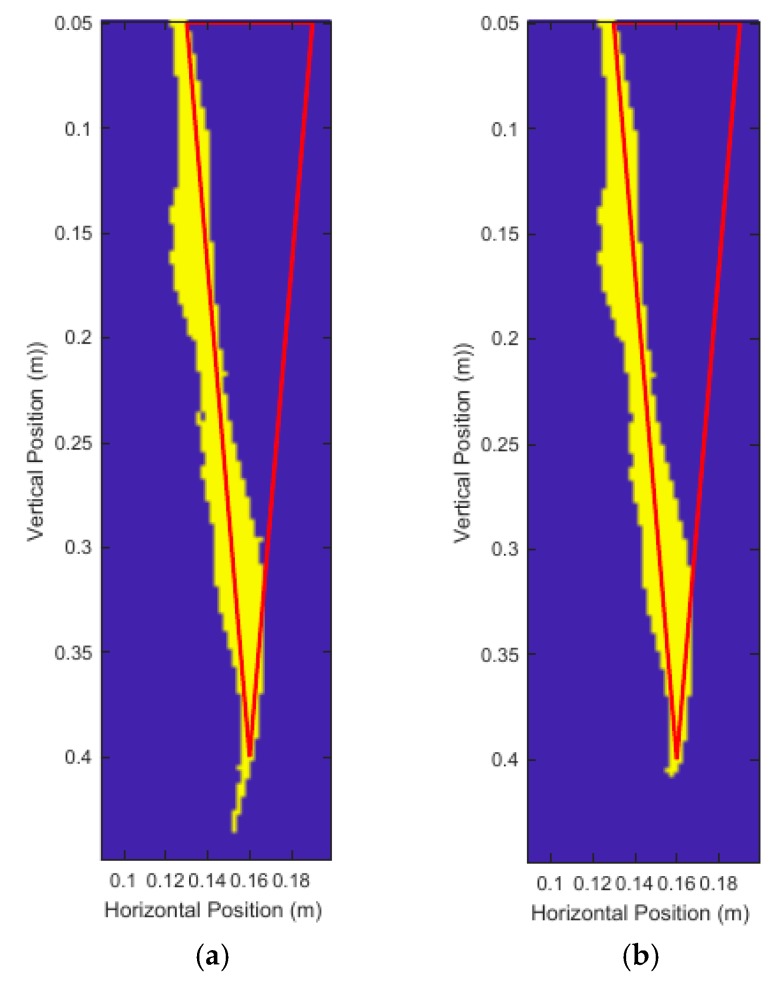
Binary masks for: (**a**) no binary erosion performed and (**b**) a binary erosion and dilation performed.

The high intensity distortion near the bottom tip of the root is a common artifact caused by the beamforming algorithm. Occasionally, the modified top-hat transformation isn’t sufficient to completely remove this ‘tail’ artifact. Once a quantized image is formed, we can use a binary erosion on the image to remove small distortions based on size and shape only. A subsequent binary dilation is also performed to restore any lost edges on the larger regions remaining after the binary erosion. Figure 24 shows the results of a binary erosion and dilation of 1 mm diameter on Figure 23b.

The binary erosion significantly improved the measurements of the root by removing the ‘tail’ artifact almost entirely. The image with no binary erosion had a depth of 38.97 cm and average root diameter of 2.87 cm. The image with a binary erosion and dilation had a depth of 36.19 cm and average root diameter of 2.97 cm. The true measurements were a 35.00 cm depth and 3.00 cm average root diameter.

Overall, morphological transformations are essential tools for refining the images produced by DAS beamforming, but careful selection of the parameters based on prior knowledge of the root (for example, type and approximate size for the plant age) is needed to improve results.

#### 3.1.5. Image Quantization Parameter Adjustment Results

High intensity regions in the image produced by the delay-and-sum beamforming algorithm corresponded to the location of reflecting materials in the media. If these high intensity regions are relatively localized to the surface of the root, and there are not many other regions of high intensity in the image, then the energy histogram and cumulative energy histogram will show that most of the energy lies in higher intensity pixels.

The binary mask will keep a certain percentage of energy in the image, starting from the higher intensity pixels. Appropriate selection of this percentage is crucial to retaining information on the root while removing noise in high noise images. For images with low noise, any percentage between 30% and 60% will perform well in measurements. For higher noise images, a larger percentage is needed to prevent the removal of root information. The high noise image as shown in Figure 21b shows that distortions and artifacts dominate the image. To handle this, a higher percentage of energy is retained in the mask to ensure that all the root information is kept.

**Figure 25:**
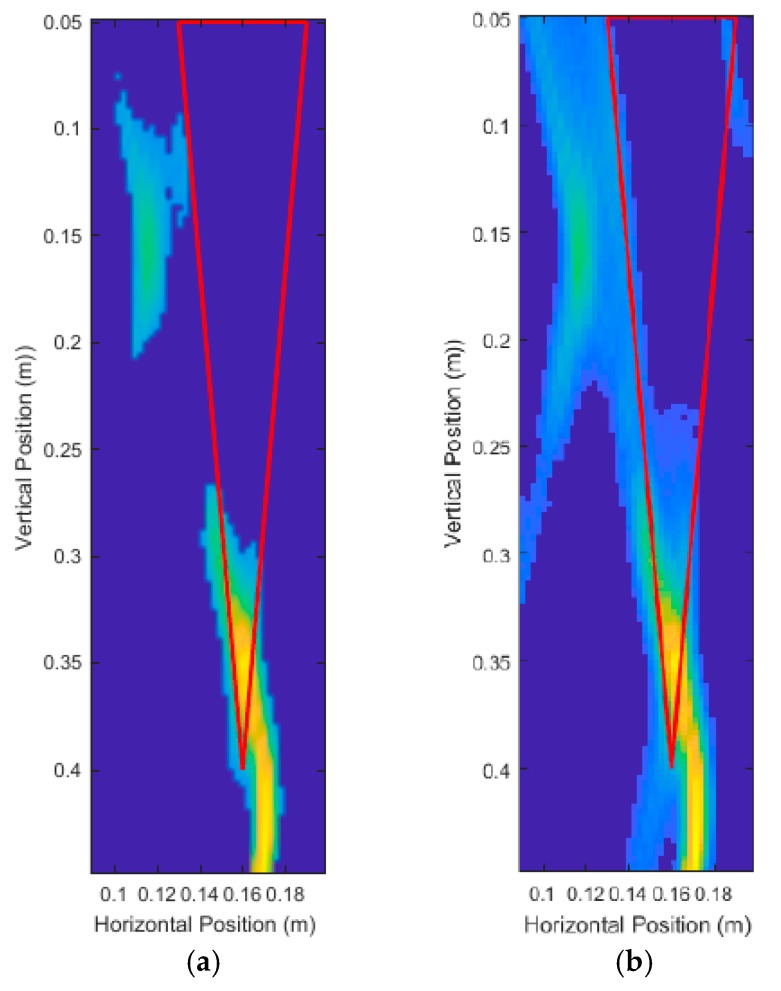
Comparison of a high noise images with: (**a**) 35% percent energy retained and (**b**) 70% percent energy retained.

Figure 24 shows that retaining 35% keeps the artifacts caused by other scatterers in the soil. Since the noise in the image dominates the desired root reflections, we cannot rely on assuming that the root will be the best reflector in the soil. Instead, more energy must be retained, in this case 70%, to keep the desired root information. Assuming that the actual location of the root is relatively unknown, it is still very difficult to isolate the root surface. Note that Figure 25a,b were obtained by using the Hadamard Product of T(x,y) and Q(x,y). As a rule of thumb, as briefly mentioned in Section 2.4.2 on energy histograms, if the 50% energy threshold is on the lower half of the range of 0 to 255 for an 8-bit grayscale image, there are likely many other scatterers in the soil and a larger percentage of energy should be retained to ensure the root information is kept in the quantized image. Exact 50% energy threshold values which the produced images should have to indicate low noise are difficult to determine and vary based on the physical parameters of the system under test.

### 3.2. Experimental Trial Results

**Figure 26:**
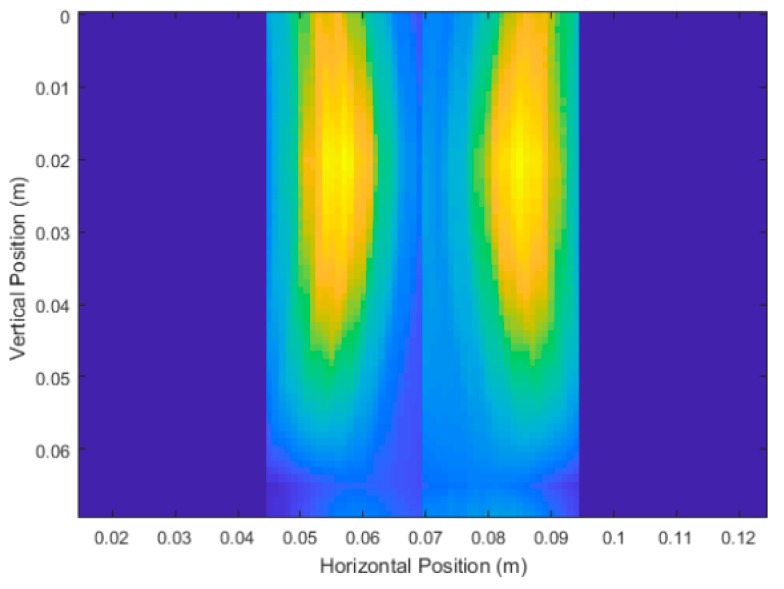
Unprocessed DAS beamforming results of Carrot 1 Side 1 with vertical scans taken at 0° and 180°, depth measurements after processing of 5.7 cm and 5.8 cm respectively. Average diameter after processing measured to be 2.85 cm.

**Figure 27:**
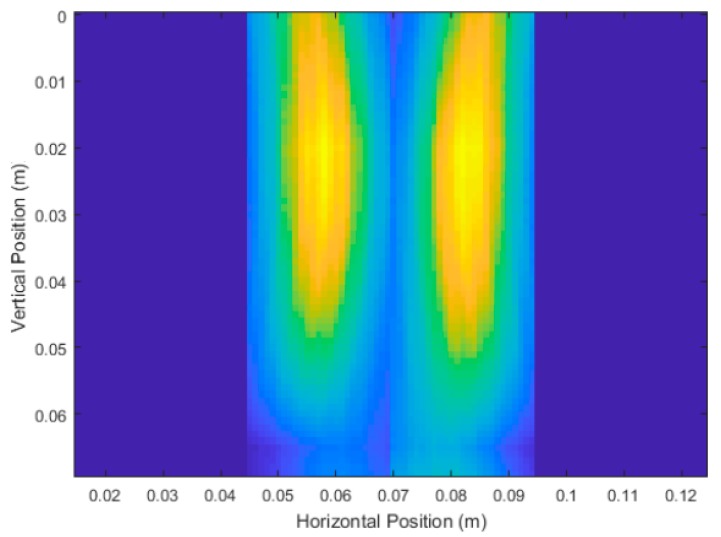
Unprocessed DAS beamforming results of Carrot 1 Side 2 with vertical scans taken at 90° and 270°, depth measurements after processing of 5.7 cm and 6.4 cm respectively. Average diameter after processing measured to be 2.36 cm.

Figure 26 and Figure 27 show the unprocessed DAS beamforming results for Carrot 1, which was measured to have a depth of 6.3 cm and an average diameter of 2.1 cm. The scans at 180° and 270° were flipped and concatenated with 0° and 90°, respectively, to create a cross-sectional image of the scanned root in the pot for easier viewing. Each of the images were still processed individually for measurements. Similar to the simulated experiments, the large size of the high intensity areas made determination of the location of the root fairly uncertain relative to the root’s overall size. This caused issues with determining finer details in the root itself. Using the developed image processing and analysis methods from the simulations, we measured an average depth measurement of 5.9 cm and an average diameter of 2.6 cm.

**Figure 28:**
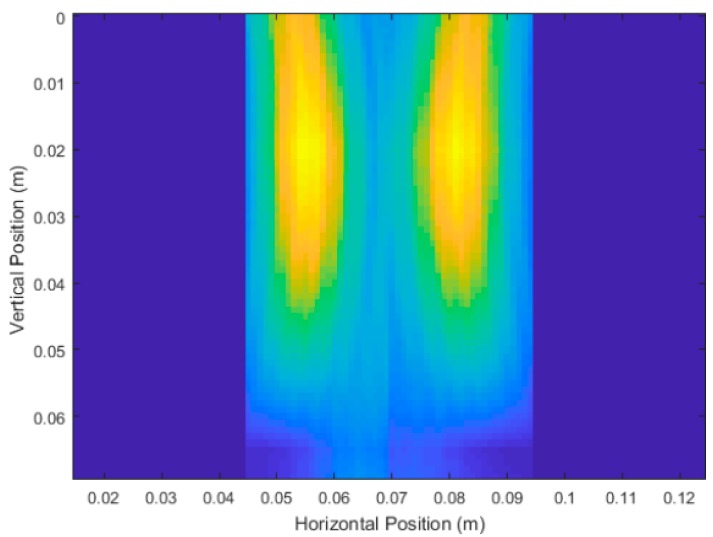
Unprocessed DAS beamforming results of Carrot 2 Side 1, with vertical scans taken at 0° and 180°, and depth measurements after processing of 5.5 cm and 5.1 cm respectively. Average diameter after processing was measured to be 2.40 cm.

**Figure 29:**
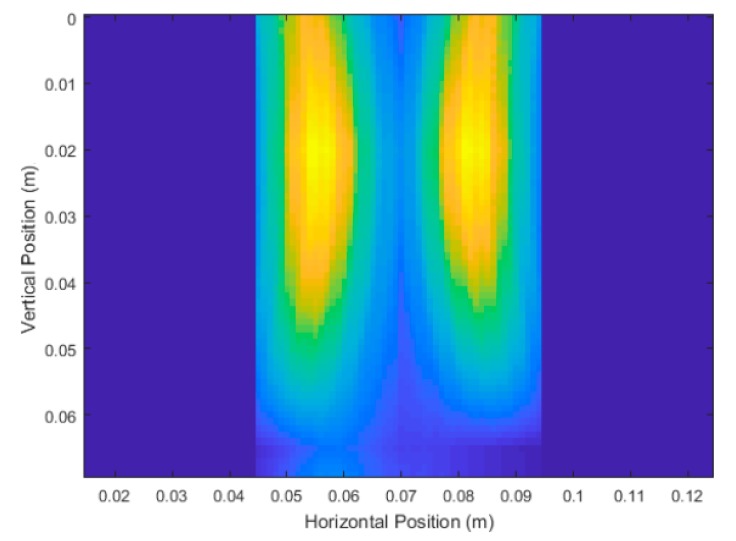
Unprocessed DAS beamforming results of Carrot 2 Side 2, with vertical scans taken at 90° and 270°, and depth measurements after processing of 5.6 cm and 5.3 cm respectively. Average diameter after processing was measured to be 2.63 cm.

Figure 28 and Figure 29 show the results for Carrot 2, which was measured to have a depth of 5.7 cm and an average diameter of 2.1 cm. These set of scans suffered from the same issues as the other root, but the system showed consistency in its measurements. We have an average depth measurement of 5.4 cm (averaged across the four produced images) and an average diameter measurement of 2.5 cm. 

Table 3 summarizes the experimental trial results, as well as the ground truth results. The ground truth results were determined by measuring RGB images of the carrots before they were buried in the pot. Note that the measured depths for all tests were consistently lower than the true depth. This suggests difficulties in detecting the tips of the carrots in the soil and resulted in larger average diameter measurements.

### 3.3. Experimental Trial Replication Results

Next, the experimental trials were replicated in simulation. Simulating the circumstances for the experimental trials was needed to accomplish two crucial goals. The first goal was to verify the accuracy of the simulation methodology and the simplifications that were made to reduce simulation complexity. The second goal was to examine faults in design and seek sources of improvement for the experimental trials. 

**Figure 30:**
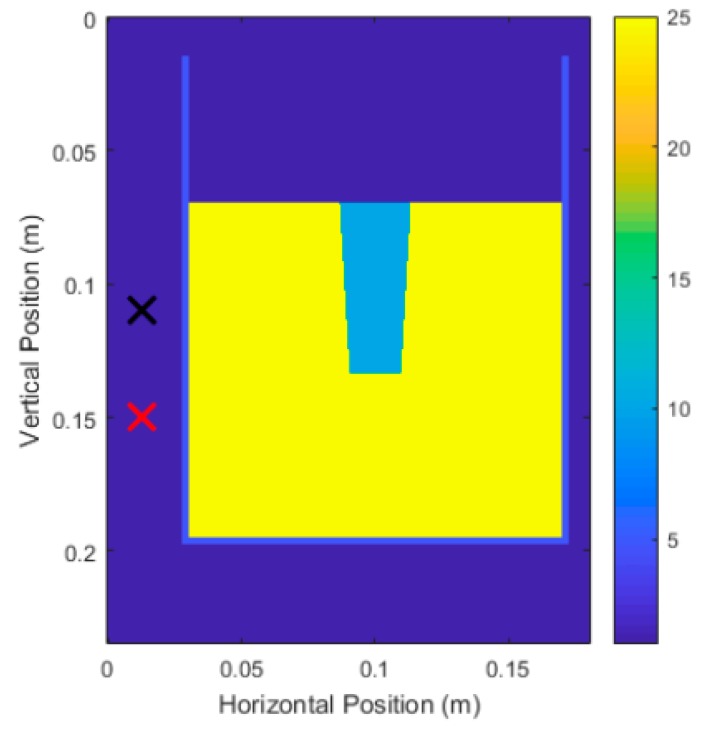
Physical and electrical parameters of system under test which models the experimental trials.

Figure 30 shows an example of the physical and electrical parameters of the system under test for simulating Carrot 1, Side 1 from the experimental trials. $\epsilon_{r,soil}=25$ is selected and $\epsilon_{r,root}=10$ is selected. The soil relative permittivity was selected using trial and error to yield the best measurements, and the selected value aligns with the higher end of the relative permittivity found in literature for dry soils [32,39]. The root relative permittivity was selected to provide adequate contrast between root and soil. The transmitter and receiver are separated by 4 cm to simulate the bi-static set-up of the P410 antenna. The height used for the DAS beamforming algorithm was the height of the transmitter for the experimental trials. The top marked ‘x’ was the transmitter location while the bottom marked ‘x’ is the receiver location. The transmitter and receiver were positioned 1.5 cm from the surface of the pot. 10 scan positions were made, starting at 2 cm depth from the soil and ending at 11 cm depth. The root itself was modeled by taking the diameter at the top and diameter at the bottom, and linearly interpolating the shape of the root in between top and bottom.

**Figure 31:**
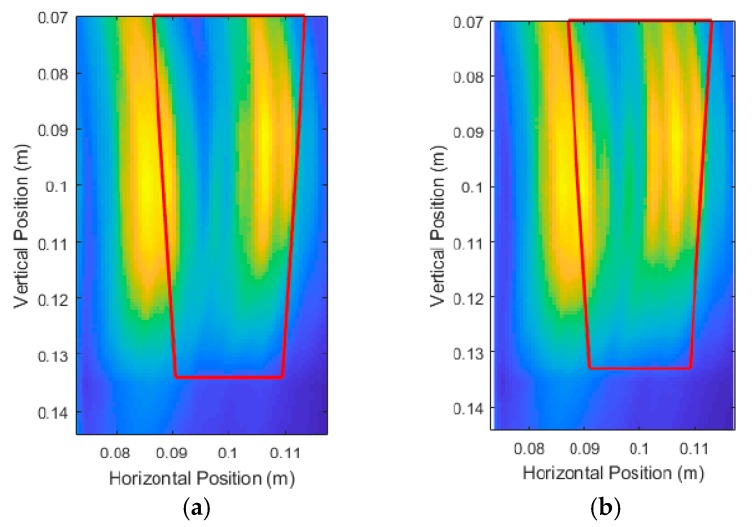
Unprocessed DAS beamforming images of the experimental replication trials for: (**a**) Carrot 1, Side 1 and (**b**) Carrot 1, Side 2.

**Figure 32:**
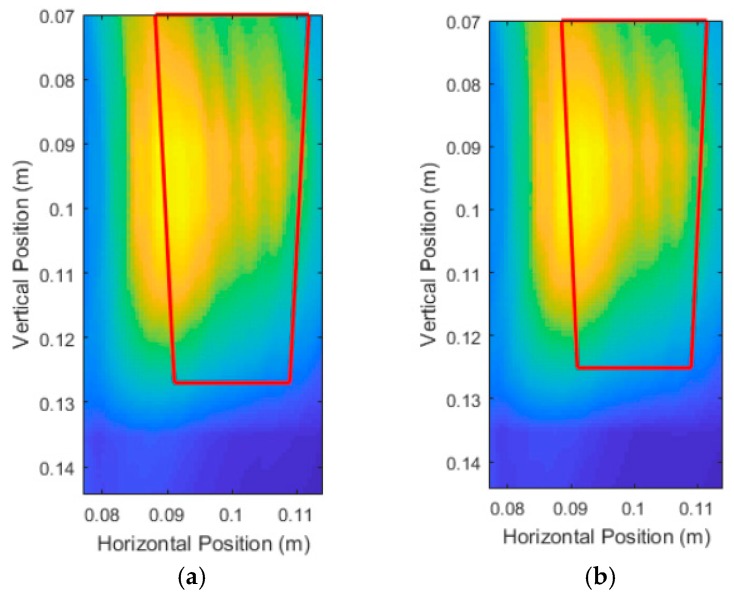
Unprocessed DAS beamforming images of the experimental replication trials for: (**a**) Carrot 2, Side 1 and (**b**) Carrot 2, Side 2.

Figure 31 shows the results of the simulations for Carrot 1. Note that the DAS beamforming was done for areas past the center of the pot to check if the back surface of the root interfered with the front surface of the root. For Carrot 1, the back surface reflections did not interfere with the front surface. Figure 32 shows the results for Carrot 2; however, there was some interference due to the differences in the size and shape of the root. This shifted the center of the area towards the center of the pot, which coincidentally was beneficial to the measurement algorithm, but only if this interference was known to be present. To compensate for this interference, instead of removing half the area of the intensity area, we removed one-third of the area for better measurements.

Ignoring the area past the center of the pot (which was 10 cm in the horizontal position of the simulation grid), we arrived at very similar qualitative results to the experimental trials. This indicates that the simplifications made in our simulations had limited impact on the results, at least for the current hardware and set-up limitations. Table 4 and Table 5 shows that the measurements between the experimental trials and the simulated recreation were also similar.

A large limitation on the resolution of the P410 device is the duration of the emitted UWB pulse. Increasing the bandwidth and the frequency of the UWB pulse resulted in a much shorter UWB pulse emitted. This improved the quality of the DAS beamforming images significantly, since the size of the window used could be reduced, resulting in more localized regions of high intensity in the final DAS beamforming image. 

**Figure 33:**
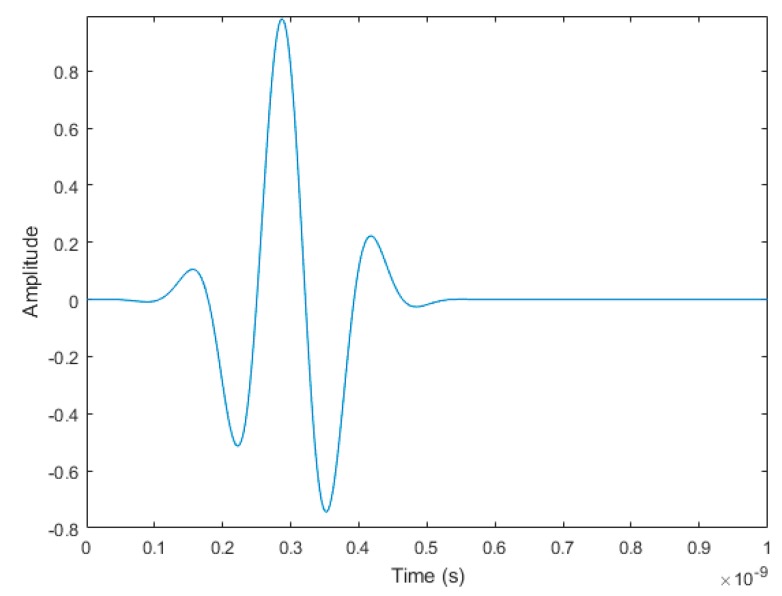
2 GHz to 12 GHz waveform used in high frequency simulations.

The frequency of the source pulse was increased to be 2 GHz to 12 GHz. Figure 33 shows the pulse to be injected in the simulations. Note that the duration of this pulse was significantly shorter than all previous tests, at about 0.4 ns.

**Figure 34:**
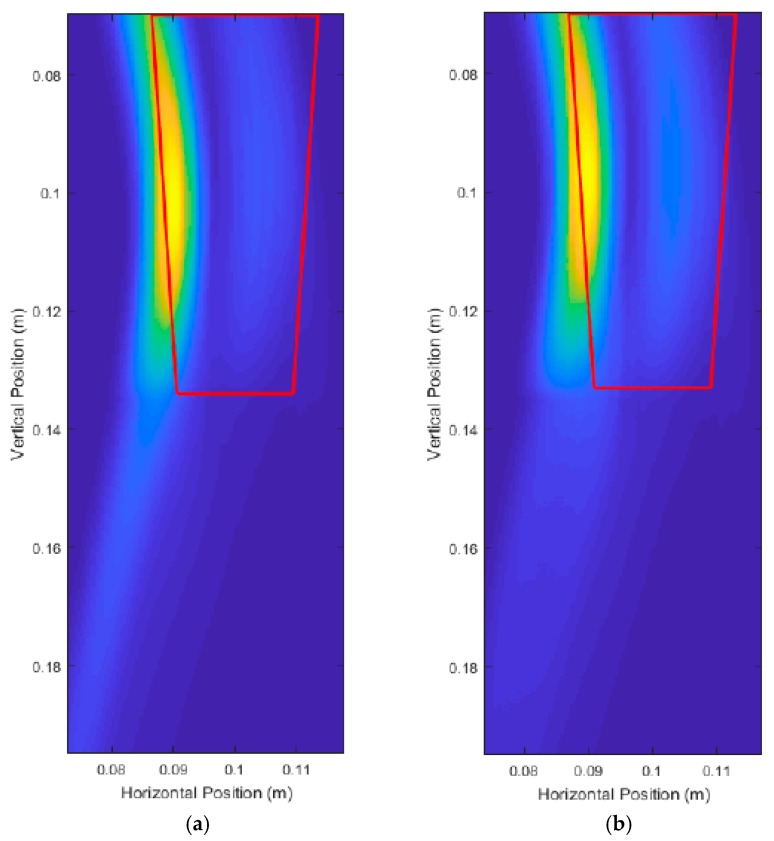
Unprocessed DAS beamforming images using a high frequency source pulse for: (**a**) Carrot 1, Side 1 and (**b**) Carrot 1, Side 2.

Carrot 1 was simulated again with the waveform depicted in Figure 33, and with the same electrical and physical parameters as before. Figure 34 shows the results of increasing the frequency and bandwidth of the source pulse. The high intensity regions were much more localized around the surface of the root, resulting in much better measurements by the image analysis module as shown in Table 6 and Table 7.

## 4. Discussion and Conclusions

An ultra-wideband system was designed to collect, process, and analyze reflection data on potted roots in this paper. Three main modules were designed: a Data Acquisition module, a Data Processing module, and an Image Processing and Analysis module. The Data Acquisition module sought to collect data on root reflections. Initially, finite-difference time-domain simulations were done to model the electromagnetic interactions of an emitted 3.1 GHz to 5.3 GHz ultra-wideband pulse with a pot and root system under test. A non-contact, mono-static, single transceiver synthetic aperture radar set-up was used to scan the pot and root model. The root was simulated to be a taproot, a type of root which consists of a large primary root. Previous studies using ultra-wideband frequencies use a similar set-up to scan breast tissue for tumors and whole bodies for concealed weapons. The only major study using an ultra-wideband system to image roots also used the same set-up. The Data Processing module received the reflection data from the Data Acquisition module and uses delay-and-sum beamforming to create an image. Delay-and-sum beamforming uses known physical and electrical parameters to estimate the propagation time of the ultra-wideband pulse to go from transmitter, to an arbitrary imaging point, and back to a receiver. This information was then used to create a mapping of backscattered energy as an image. The Image Processing and Analysis module is responsible for processing and analyzing the image formed by the data processing module to detect, and to measure the surface location of the root that was scanned. This module measured the root depth and average root diameter in an unsupervised fashion. The error in these two measurements were the primary metrics the system was evaluated upon. 

A non-contact, bi-static, synthetic aperture radar set-up was used for experimental trials. For one test, the carrot was buried approximately 6.3 cm deep and had an average diameter of 2.2 cm. For the second test, the carrot was buried approximately 5.6 cm and had an average diameter of 2.1 cm. The images produced from the experimental trials performed reasonably well, but the quality was still quite poor relative to the simulated results on larger roots. For the first test, the system measured approximately a 5.9 cm depth (true depth of 6.3 cm) and a 2.6 cm average diameter (true diameter of 2.2 cm). For the second test, the system measured approximately a 5.4 cm depth (true depth of 5.6 cm) and a 2.5 cm average diameter (true diameter of 2.1 cm).

The simulated trials provided information on essential system parameters and how those parameters affected the quality of the image and measurements. These parameters significantly impacted the quality of the results of the UWB imaging system and interacted with each other in complex ways that need to be understood to design an optimal system. The most important system parameter to measure and design for were the soil and root relative permittivities. There was an ongoing need for research into measuring and understanding the complex and highly dynamic conditions of root and soil electrical parameters for use in non-destructive root imaging. The soil in the experimental trials was maintained to be as dry as possible for consistent soil conditions. In application, constraints on the soil conditions will interfere with the growth of the root to be imaged. Research into determining optimal soil conditions for imaging using UWB frequencies, measuring the relative permittivity of the soil in situ, and methods of manipulation of the soil conditions to maximize image quality are all topics which will greatly improve this UWB imaging method. More knowledge on the soil conditions allows for better design of the imaging system and less constraints on how the soil needs to be maintained. In future works, applications of other low-cost non-destructive sensors and methods to measure the conditions of the plant roots and soil will be critical for maximizing the quality of the UWB imaging method. Other low-cost non-destructive methods of root characterization such as electrical impedance spectroscopy (EIS), which non-destructively estimates root biomass through root and soil using impedance measurements [12,41,42,43], may provide insight into important root and soil parameters, which may be used to improve the UWB imaging system. As such, a multi-sensor approach in future works should be considered to maximize the quality of the images produced by the system.

Another important system parameter which affected the quality of the image and measurements was the frequency range of the device. Section 3.3 shows that there can be significant improvements in using a 2 GHz–12 GHz pulse over the 3.3 GHz–5.1 GHz pulse that the P410 device provided. Care must be taken to account for potential electromagnetic phenomena which may impact the results of the imaging system at these higher frequencies. Care must be taken when increasing frequency, as any anisotropy or dispersiveness in the soil may cause more pronounced effects on the imaging results at higher frequencies. Theoretically, the resolution of the system may be improved by increasing the bandwidth of the system and will be able to image roots 1 mm in diameter or thinner if the signals are conditioned appropriately to maximize the signal-to-noise ratio.

A DAS beamforming was used in all produced images in this paper; however, other beamforming algorithms are relatively unexplored for this application in root imaging and are a topic of research for the future. More sophisticated algorithms which involve modifying the steering vector include Kirchhoff migration, migration by de-convolution, and minimum variance beamforming. Optimization of these beamforming algorithms rely on having knowledge of the material properties of the system under test, once again emphasizing the need for more sophisticated devices to measure soil properties in real time.

This work explored the feasibility of UWB technology as a low-cost and portable solution to non-destructively measure potted taproot plants. In the end, the results of this work determined many important design decisions needed to the development of an UWB system for non-destructively imaging roots. The success of the system itself is heavily dependent on many parameters and variables such as soil conditions, root sizes, and hardware limitations, as analyzed in this paper.

## Figures and Tables

**Table 1:** Measured root depths and average root diameters for images produced by varying the number of scanning positions.

Number of Scans	Measured Root Depth (cm)	Error in Depth Measurement (cm)	Measured Average Root Diameter (cm)	Error in Average Diameter Measurement (cm)
6	30.25	−5.75	3.85	+0.85
12	36.14	+1.14	3.14	+0.14
21	34.69	−0.31	3.29	+0.29

**Table 2:** Measured root depths and root average diameters for images produced by varying the number of scanning positions.

Root Depth (cm)	Percent Error in Depth Measurement (%)	Average Root Diameter (cm)	Percent Error in Average Root Diameter Measurement (%)
25.00	4.3	2.00	15.6
15.00	13.5	1.50	26.4
10.00	30.4	1.50	18.7
5.00	112.8	1.00	37.5

**Table 3:** Summary of depth and average diameter measurements on the experimental trials.

Carrot Number and Side	Depth Measurement (cm)	True Depth (cm)	Diameter Measurement (cm)	True Diameter (cm)
Carrot 1, Side 1	5.75	6.29	2.85	2.32
Carrot 1, Side 2	6.05	6.26	2.36	2.13
Carrot 2, Side 1	5.45	6.61	2.40	2.10
Carrot 2, Side 2	5.30	5.51	2.63	2.08

**Table 4:** Summary of depth measurements on the experimental trials and simulated recreation.

Carrot Number and Side	Experimental Depth Measurement (cm)	Simulated Depth Measurement (cm)	True Depth (cm)
Carrot 1, Side 1	5.75	6.13	6.29
Carrot 1, Side 2	6.05	5.93	6.26
Carrot 2, Side 1	5.45	5.53	5.61
Carrot 2, Side 2	5.30	5.33	5.51

**Table 5:** Summary of depth and average diameter measurements on the experimental trials.

Carrot Number and Side	Experimental Average Diameter Measurement (cm)	Simulated Average Diameter Measurement (cm)	True Diameter (cm)
Carrot 1, Side 1	2.85	2.64	2.32
Carrot 1, Side 2	2.36	2.26	2.13
Carrot 2, Side 1	2.40	2.33	2.10
Carrot 2, Side 2	2.63	2.34	2.08

**Table 6:** Summary of depth measurements on the experimental trials and simulated recreation.

Carrot Number and Side	Experimental Depth Measurement (cm)	Simulated High Frequency Depth Measurement (cm)	True Depth (cm)
Carrot 1, Side 1	5.75	6.48	6.29
Carrot 1, Side 2	6.05	6.36	6.26

**Table 7:** Summary of depth and average diameter measurements on the experimental trials.

Carrot Number and Side	Experimental Average Diameter Measurement (cm)	Simulated Average Diameter Measurement (cm)	True Diameter (cm)
Carrot 1, Side 1	2.85	2.21	2.32
Carrot 1, Side 2	2.36	2.21	2.13

**Figure A1:**
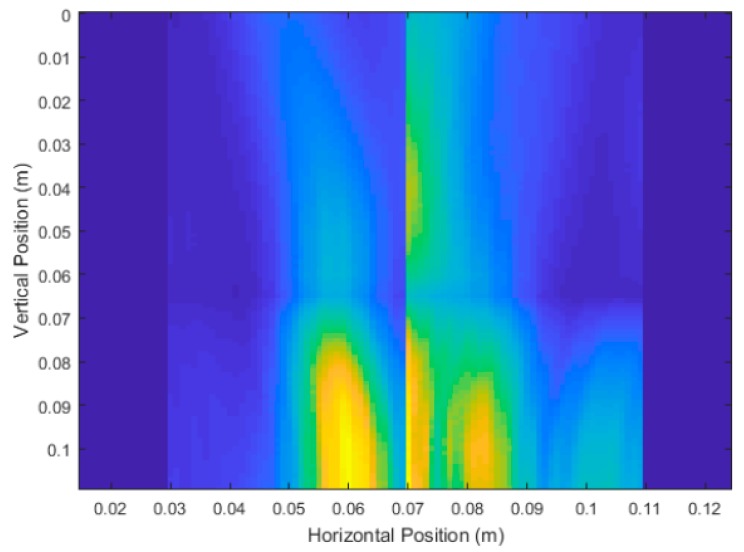
Unprocessed DAS beamforming results of a potato taken at 0° and 180°.

**Figure A2:**
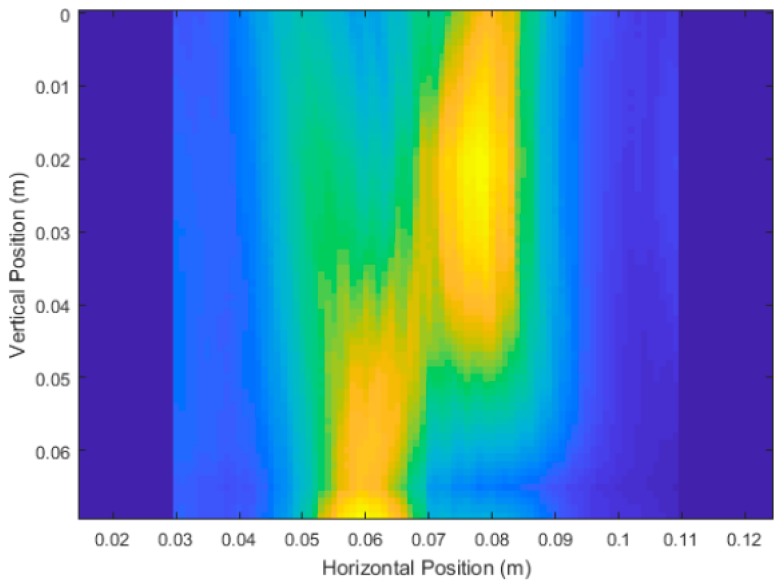
Unprocessed DAS beamforming results of a beet root taken at 0° and 180°.

**Figure A3:**
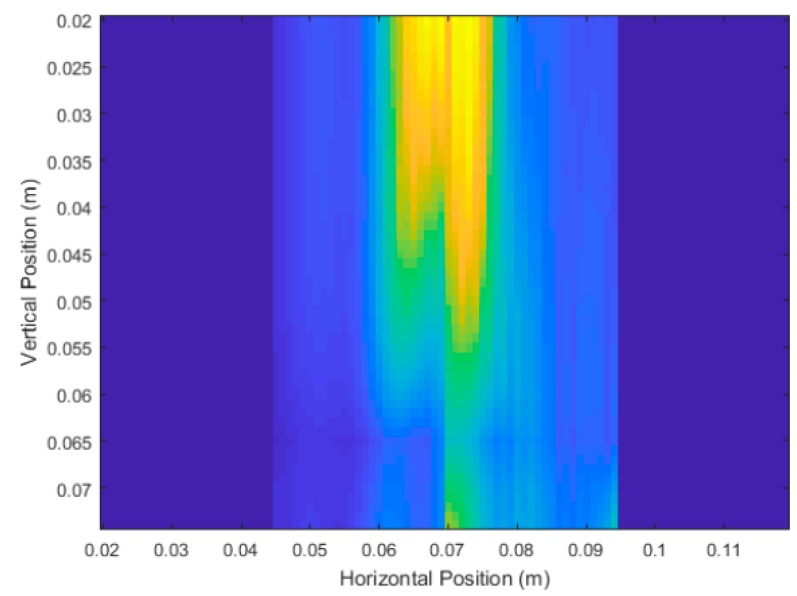
Unprocessed DAS beamforming results of carrot taken at 0° and 180°.

**Figure A4:**
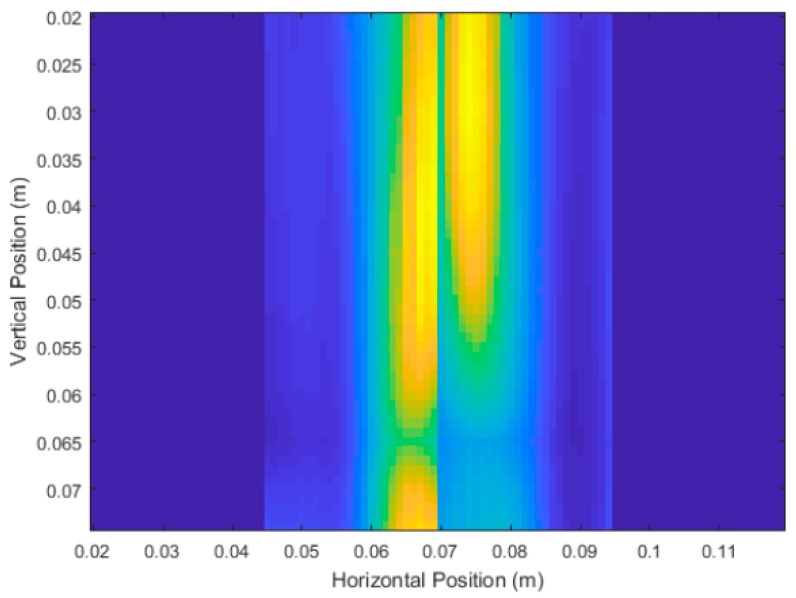
Unprocessed DAS beamforming results of a carrot taken at 45° and 225°.

**Figure A5:**
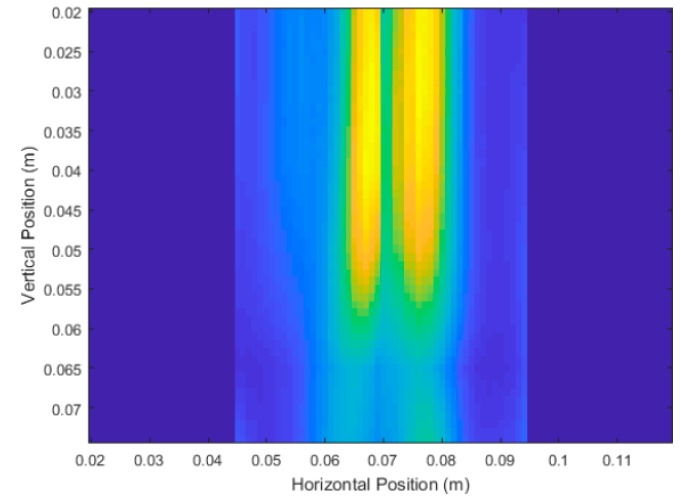
Unprocessed DAS beamforming results of a carrot taken at 90° and 270°.

**Figure A6:**
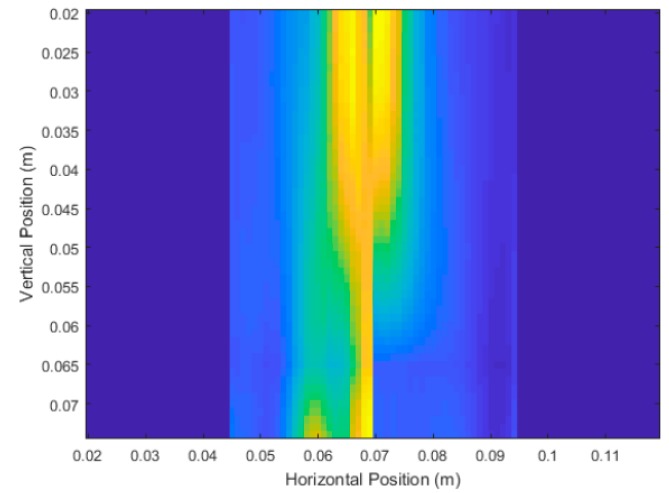
Unprocessed DAS beamforming results of a carrot taken at 135° and 315°.

## Appendix A. Additional Imaged Results on Various Taproots

Figure A1 shows the imaging results of a buried potato of approximately 9 cm depth and 4 cm average diameter. The quality is very poor, and upon brief examination reveals that the amplitude of reflections of the potato are of similar amplitude to noise in the signal. This is likely due to a poor relative permittivity contrast between soil and root and the presence of scattering heterogeneities in the soil.

Figure A2 shows the imaging results of a buried red beet with approximately 6 cm depth and 5 cm average diameter. The image taken at 0° is dominated by scatterers in the soil medium, but the outline of the beet is still somewhat present.

Figure A3, Figure A4, Figure A5 and Figure A6 contain the imaging results from various angles on a carrot of approximately 6 cm depth and 3 cm average diameter. Again, occasional artifacts arise, likely from some unwanted high energy reflections caused by heterogeneities in the soil.
